# Association of circulating leptin, adiponectin, and resistin concentrations with long-term breast cancer prognosis in a German patient cohort

**DOI:** 10.1038/s41598-021-02958-w

**Published:** 2021-12-07

**Authors:** Nadia Obi, Audrey Y. Jung, Tabea Maurer, Marianne Huebner, Theron Johnson, Sabine Behrens, Stefanie Jaskulski, Heiko Becher, Jenny Chang-Claude

**Affiliations:** 1 Institute for Medical Biometry and Epidemiology, University Medical Center Hamburg-Eppendorf, Martinistr. 52, 20246 Hamburg, Germany; 2 Division of Cancer Epidemiology/Unit of Genetic Epidemiology, German Cancer Research Center (DKFZ), Im Neuenheimer Feld 581, 69120 Heidelberg, Germany; 3 Cancer Epidemiology, University Cancer Center Hamburg, University Medical Center Hamburg-Eppendorf, Hamburg, Germany; 4 Department of Statistics and Probability, Michigan State University, East Lansing, MI 48824 USA; 5 Institute for Prevention and Cancer Epidemiology, Faculty of Medicine and Medical Center, University Medical Center, University of Freiburg, Freiburg, Germany

**Keywords:** Biomarkers, Prognostic markers, Breast cancer, Cancer epidemiology, Breast cancer, Cancer epidemiology

## Abstract

Adipokines including leptin, adiponectin and resistin have been linked to risk of obesity-related cancers potentially through low-grade chronic inflammation pathways. We aimed to assess the role of post-diagnosis circulating adipokines on long-term prognosis in a prospective breast cancer cohort. Adipokines were measured in blood collected at baseline shortly after diagnosis (2002–2005) and at follow-up (2009) from 3112 breast cancer patients enrolled in the population-based MARIE study. Half of the patients had measurements at both time-points. All-cause mortality, breast cancer specific mortality and recurrences were ascertained up to June 2015 (11 years median follow-up). Associations with time-varying adipokine concentrations overall and stratified by estrogen and progesterone receptor (ERPR) were evaluated using adjusted proportional hazard regression. At baseline (*n* = 2700) and follow-up (*n* = 2027), median concentrations for leptin, adiponectin and resistin were 4.6 and 2.7 ng/ml, 24.4 and 30.0 mg/l, 15.4 and 26.2 ng/ml, respectively. After adjustment, there was no evidence for associations between adipokines and any outcome overall. In ERPR negative tumors, highest vs. lowest quintile of adiponectin was significantly associated with increased breast cancer specific mortality (HR 2.51, 95%CI 1.07–5.92). Overall, post-diagnosis adipokines were not associated with long-term outcomes after breast cancer. In patients with ERPR negative tumors, higher concentrations of adiponectin may be associated with increased breast cancer specific mortality and warrant further investigation.

## Introduction

The adipokines, leptin, adiponectin and resistin, are lipid hormones that are predominantly produced in white adipose tissue. They have pro- and anti-inflammatory properties and play a role in low-grade chronic inflammation^1^, metabolic homeostasis^1^ and tumorigenesis^2^. Leptin has been shown to regulate immune responses and promote cell proliferation- and growth factor-related effects^3^, but mainly regulates central nervous system effects in the hypothalamus, i.e. energy intake by inhibiting hunger^4^. In states of overweight/obesity leptin sensitivity might be impaired causing an increased production^4^. Adiponectin exerts antagonistic functions to leptin; it regulates lipid metabolism, increases insulin sensitivity^5^, inhibits cell growth and cell survival^6^, and is up-regulated after weight loss^7^. Resistin is secreted from macrophages in adipose tissue and at high concentrations may induce insulin resistance and promote malignancies^8^. In spite of these characteristics, studies on obesity-related breast cancer risk yielded inconsistent results for the adipokines, partly depending on whether blood was collected long-term pre-diagnosis^9–12^ or post-diagnosis^13–15^ and on adjustment for BMI^16^. Major pre-diagnosis cohort studies found no relationships for leptin^9,11^, adiponectin^9–12^ and resistin^9,12^. However, in a recent meta-analysis, both overweight women and postmenopausal women with higher leptin concentrations were at higher risk for breast cancer^17^, and post-diagnosis case–control studies have reported higher circulating resistin to be a risk factor for postmenopausal breast cancer, which correlated with higher stage, lymph node metastasis, and negative hormone receptor status^14,15,18^.

Some studies have investigated breast cancer prognosis in relation to post-diagnosis peripheral leptin and/or adiponectin^19–25^ or resistin^26,27^ or tissue concentrations, with heterogeneous findings. Higher circulating leptin has been associated with increased recurrence and mortality for all breast cancers^20^ or only ER positive tumors^22^, while lower leptin immunostaining was related to more recurrences and poorer overall survival^28,29^. Peripheral adiponectin concentrations above the mean/median were associated with lower breast cancer specific mortality (BCM)^21^, better disease-free survival^23,24^, and lower recurrence in ER negative breast cancer only^19^, whereas expression in triple negative breast cancer was not associated with survival^30^. Circulating resistin concentrations were found to be associated with shorter^27^ or longer survival of breast cancer patients^26^, and tissue expression of resistin was related to higher all-cause mortality^31^ and hormone receptor negative disease^32^. Thus, while pre-clinical studies suggest signaling pathways of adipokines lead to tumor progression (metastatic spread) or inhibition (e.g. apoptosis), conflicting epidemiological evidence does not support clinical utility of adipokines in breast cancer management. Moreover, all cited studies used only one measurement of adipokines at baseline. We investigated the role of circulating adipokines in long-term breast cancer prognosis using measurements from two time-points around 5 years apart in a large cohort of postmenopausal breast cancer patients and examined potential associations of time-varying leptin, adiponectin and resistin with all-cause mortality, BCM and risk of recurrence. Furthermore, we addressed potential modification by hormone receptor status and BMI.

## Methods

Reporting of the present study was conducted according to the REMARK-statement^33^. A study profile is shown in supplemental Table S1 [see Supplemental information].

### Study population

We conducted a prospective cohort study with 3813 incident breast cancer patients diagnosed between January 1, 2001 and September 30, 2005 in two regions of Germany, Hamburg and Rhine-Neckar-Karlsruhe (RNK) and recruited by the population-based case–control MARIE study (Mamma Carcinoma Risk Factor Investigation). Eligible patients were aged 50–74 years, 9.3% of which were peri- and 90.7% were postmenopausal, and had a histologically confirmed diagnosis of primary invasive or in situ breast cancer^34^. Histological characteristics of the primary tumor were abstracted from pathology reports. At recruitment, a standardized personal interview provided information on pre-diagnosis lifestyle factors, morbidity and therapies. During follow-up in 2009 and in 2014/2015, medical records were checked or treating physicians were contacted to identify and validate patient reports on breast cancer treatments, occurrences of relapse, metastasis or second tumors. Information on vital status and causes of death was obtained from population registries and local health authorities. Patients were followed-up until death, emigration or last contact until the date of censoring (June 30, 2015), whichever came first.

**Figure 1:**
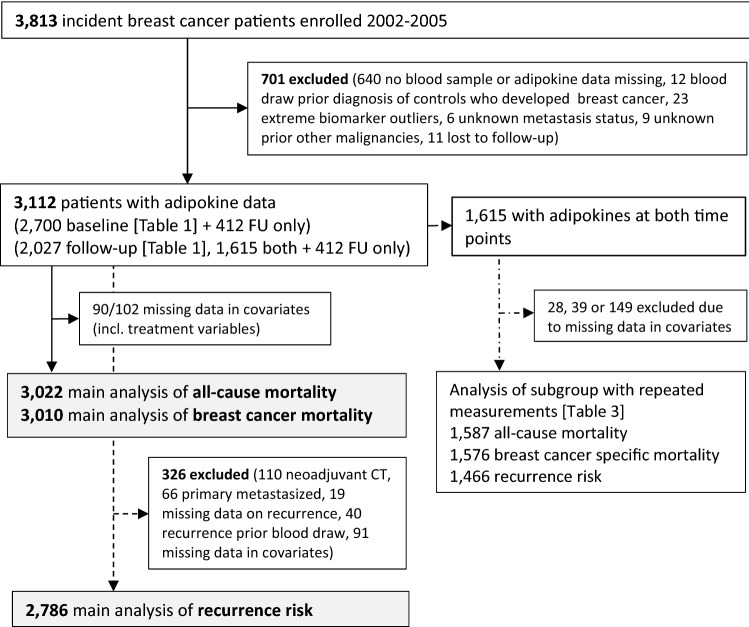
Flowchart of breast cancer patients included in the analysis according to available adipokine measurements. Analyses of all-cause mortality and breast cancer mortality/recurrence risk included different sets of covariates, therefore amount of missing values differ.

Non-fasting blood samples were collected post-diagnosis (2002–2005) at recruitment and at first follow-up (2009). 701 patients (18.4%) were excluded for unavailable blood sample, incomplete adipokine measurements or being outliers, unknown metastasis, other malignancies and loss to follow-up (Fig. 1), resulting in a final analysis population of 3112 patients. Thereof, adipokine measurements were available at both baseline and follow-up (*n* = 1615), at baseline only (*n* = 1085), or at follow-up only (*n* = 412). Outcome-specific exclusions of patients due to missing data are shown in Fig. 1.

For analysis of all-cause mortality and BCM, women with breast cancer stage 0–4 at diagnosis were included. For analysis of recurrence-free interval (stages 0–3c included), patients with metastatic and neoadjuvant chemotherapy-treated tumors were excluded to assure patients were free of metastasis, since stage has not been assigned in the latter. Recurrences were defined as incident regional metastasis, second tumors, ipsi- and contralateral, and any distant metastasis. In subgroup analysis, associations with adipokines were stratified by estrogen/progesterone receptor status (ERPR), excluding in situ tumors and tumors treated with neoadjuvant chemotherapy as ERPR was missing. Potential modification by BMI and age was assessed as well.

### Laboratory measurements

At recruitment, non-fasting serum (in RNK) and plasma (in Hamburg) were collected a median time after diagnosis of 4.3 months (IQR 0.5–13.3 months). At follow-up, non-fasting serum was collected. Following blood collection at baseline and follow-up, all samples were stored in aliquots at −80 °C until measurement in 2016. Leptin was measured with Prototype Customer Assay using the MesoScale Discovery (MSD) Electrochemiluminescence platform with multiplex capacity. Adiponectin and resistin were measured as singleplex also using MSD. The assays consisted of 64 × 96-well-plates (batches) each containing eight standards and two quality control samples in duplicate and 76 unknowns. Intra- and inter-batch CVs were 3.5% and 3.3% for leptin, 2.9% and 10.9% for adiponectin, and 2.3% and 9.8% for resistin, respectively. Standard curves were normally distributed for all biomarkers. Values above fit curve range as well as extreme outliers were set to missing.

### Statistical analyses

Median (IQR) baseline and follow-up concentrations of the three adipokines are presented for all covariates. Biomarkers at both time points and BMI at baseline were initially examined with partial Spearman’s correlation analysis controlled for region. Median follow-up time was calculated using reverse Kaplan–Meier.

Potential associations between the adipokines and all-cause mortality or BCM/recurrence were assessed using a delayed-entry proportional hazards regression model with time since diagnosis as underlying time variable. Observation time is date of first blood draw until date of event or end of follow-up, whichever came first. If baseline and follow-up adipokine measurements were both available, these were included as time-varying covariates, changing at date of second blood draw (otherwise concentrations were constant over time from the starting point). For hazard ratio estimation, measurements for leptin, adiponectin and resistin were each log2-transformed in the first model, indicating a doubling of the original concentration. In a second model, quintiles of each baseline adipokine concentration were utilized as exposure, whereby follow-up adipokine measurements were categorized into quintiles with baseline boundaries. We mutually adjusted all models for all adipokines. The distribution of the adipokines are given in Supplemental Figure S1.

Covariates were selected due to putative or known association with exposure and outcome. Basic models were adjusted for age (continuous), study region, and time between first blood draw and diagnosis (categories). All final models additionally included categorized BMI as a confounder, tumor size, nodal status, metastasis, grade, ERPR, prior other tumors, menopausal hormone therapy, mode of detection, and leisure time physical activity at age 50 (see Table 1 and Supplementary Table S2 for categories). In situ tumors and neoadjuvant chemotherapy were defined as separate categories of tumor-related variables. To account for treatment effects on baseline adipokine levels, a covariate indicating time of blood draw in relation to chemotherapy, i.e. whether patients had no chemotherapy or had their blood draw before, during or after chemotherapy (no chemotherapy = reference), was also included. Individual baseline and follow-up samples were measured in different batches, therefore batch was included as a random effect in all models. Models for all-cause mortality additionally comprised known risk factors, i.e. previous cardiovascular diseases (CVD), diabetes, smoking, and alcohol consumption at diagnosis. In the analysis of BCM and risk of recurrence, specific therapies that may interact with adipokines were added, i.e. radiotherapy, combined Her2 status/trastuzumab, tamoxifen/aromatase inhibitor treatment. Since the overall proportion of missing values in covariate sets did not exceed 5%, a complete case analysis was performed.

**Table 1:** Characteristics of the MARIE study patient population by adipokine concentrations at baseline and follow-up.

	Baseline	FU	Leptin (ng/ml)				Adiponectin (mg/l)				Resistin (ng/ml)			
			Baseline		FU		Baseline		FU		Baseline		FU	
	*N*	*N*	Median	IQR	Median	IQR	Median	IQR	Median	IQR	Median	IQR	Median	IQR
Total	2700	2027	4.6	(2.2, 9.2)	2.7	(1.3, 5.4)	24.4	(16.4, 36.0)	30.0	(20.1, 44.0)	15.4	(8.9, 26.2)	26.2	(14.6, 43.4)
Age														
50 to < 60 years	943	750	4.1	(1.9, 8.7)	2.6	(1.1, 5.0)	23.5	(15.7, 33.7)	30.0	(19.5, 42.5)	16.2	(8.9, 26.9)	27.5	(15.5, 45.4)
60 + years	1757	1277	4.9	(2.3, 9.4)	2.8	(1.4, 5.6)	24.9	(16.7, 36.8)	30.0	(20.4, 45.1)	15.1	(8.9, 26.0)	25.4	(14.2, 41.8)
Region														
Hamburg	1179	1003	5.3	(2.5, 10.3)	2.3	(1.1, 4.4)	24.2	(16.3, 35.2)	28.2	(18.8, 40.9)	9.0	(6.3, 13.4)	31.3	(20.5, 48.1)
RNK region	1521	1024	4.1	(2.0, 8.3)	3.2	(1.6, 6.4)	24.6	(16.5, 36.5)	31.9	(21.4, 47.5)	22.6	(15.0, 35.7)	19.3	(10.7, 36.9)
BMI at baseline														
< 22.5 kg/m^2^	591	448	1.8	(0.9, 3.1)	1.2	(0.7, 2.3)	30.4	(21.7, 43.7)	38.1	(24.8, 52.8)	13.5	(8.1, 24.5)	25.1	(15.2, 41.2)
22.5- < 25 kg/m^2^	656	552	3.3	(1.9, 6.0)	2.1	(1.2, 3.7)	25.9	(17.5, 37.0)	31.4	(21.1, 43.4)	14.2	(8.1, 23.8)	25.6	(15.2, 42.1)
25- < 30 kg/m^2^	992	736	6.0	(3.6, 9.9)	3.7	(2.2, 6.4)	22.6	(15.7, 33.7)	27.4	(18.9, 40.8)	16.3	(9.1, 27.5)	26.3	(14.1, 42.5)
30 + kg/m^2^	458	290	11.9	(6.9, 19.9)	7.2	(3.9, 13.0)	19.7	(13.7, 27.7)	24.1	(16.6, 36.6)	18.8	(11.0, 32.7)	29.6	(15.2, 50.4)
Tumor size														
T1 < 2 cm	1399	1173	4.1	(2.0, 8.5)	2.5	(1.2, 5.1)	25.5	(16.9, 36.5)	29.2	(19.4, 43.4)	15.0	(8.5, 25.6)	24.4	(13.3, 41.6)
T2 2–5 cm	864	602	5.1	(2.5, 10.2)	3.1	(1.5, 6.0)	23.0	(16.2, 34.5)	30.7	(20.4, 44.2)	15.6	(8.9, 27.5)	28.6	(17.1, 46.3)
T3 > 5 cm	91	46	5.8	(2.7, 12.9)	3.0	(1.5, 6.2)	20.0	(13.7, 30.8)	34.3	(22.2, 43.6)	18.4	(10.5, 32.0)	31.7	(19.1, 45.7)
T4 (infiltration skin/chest wall)	66	23	5.0	(2.6, 11.3)	3.7	(2.3, 9.0)	22.3	(14.5, 30.5)	34.9	(19.4, 45.6)	14.9	(8.9, 33.5)	33.8	(19.7, 46.4)
Neoadjuvant CT	106	51	4.7	(1.9, 10.0)	3.3	(2.1, 6.9)	25.1	(16.7, 41.2)	31.8	(23.7, 46.7)	17.9	(12.6, 32.9)	28.5	(21.0, 51.9)
In situ	169	131	3.8	(2.2, 8.4)	2.1	(1.1, 4.5)	25.9	(16.1, 40.9)	30.4	(21.2, 47.2)	13.2	(9.0, 24.2)	26.0	(16.2, 44.9)
Nodal status														
N0	1643	1333	4.6	(2.2, 9.2)	2.8	(1.3, 5.6)	24.5	(16.5, 36.3)	29.2	(19.2, 42.7)	14.7	(8.5, 25.4)	23.8	(12.7, 40.8)
N1 (1–3)	540	386	4.5	(2.0, 9.0)	2.6	(1.1, 4.9)	24.5	(17.0, 35.0)	32.2	(20.9, 46.0)	16.1	(8.7, 27.5)	30.1	(18.0, 45.6)
N2 (4–9)	144	91	5.0	(2.4, 10.9)	3.0	(1.4, 5.6)	21.6	(15.0, 31.8)	32.0	(22.2, 45.2)	17.6	(10.2, 26.4)	35.0	(24.0, 46.3)
N3 (10 +)	94	35	5.4	(1.9, 10.5)	3.4	(2.0, 9.8)	24.0	(16.2, 32.8)	41.2	(29.4, 56.0)	22.1	(12.9, 37.8)	40.1	(23.7, 56.9)
Metastasis														
No	2357	1834	4.7	(2.2, 9.3)	2.7	(1.3, 5.5)	24.2	(16.4, 35.5)	29.8	(19.6, 43.5)	15.2	(8.7, 26.0)	26.0	(14.3, 43.2)
yes	68	11	4.0	(1.8, 8.9)	2.1	(0.7, 4.9)	24.0	(15.7, 36.1)	36.3	(13.1, 55.0)	22.7	(11.8, 40.4)	37.4	(30.0, 46.7)
Grading														
G1	465	398	4.5	(2.0, 8.5)	2.7	(1.4, 5.4)	26.7	(18.2, 38.0)	29.8	(20.0, 43.8)	13.5	(8.0, 23.3)	23.3	(12.7, 38.0)
G2	1302	1000	4.6	(2.2, 9.3)	2.7	(1.2, 5.6)	24.0	(16.3, 35.2)	29.3	(19.2, 42.8)	15.5	(9.0, 26.7)	25.5	(13.7, 43.4)
G3	645	438	4.8	(2.2, 9.7)	2.8	(1.4, 5.5)	22.7	(15.7, 33.7)	31.2	(20.4, 44.7)	16.5	(9.3, 28.3)	30.1	(18.4, 46.0)
Hormone receptor status														
ER + /PR +	1676	1317	4.9	(2.3, 9.4)	2.8	(1.3, 5.6)	23.9	(16.3, 35.2)	29.3	(19.6, 43.2)	15.3	(8.8, 26.8)	24.9	(13.3, 43.0)
ER + /PR- or ER-/PR +	401	293	4.1	(1.9, 8.4)	2.4	(1.1, 4.8)	25.5	(17.4, 36.6)	30.8	(19.4, 44.2)	15.2	(8.7, 26.3)	27.9	(15.5, 42.8)
ER-/PR-	347	235	4.0	(2.1, 9.4)	2.7	(1.3, 5.5)	24.3	(16.6, 35.3)	31.7	(21.0, 45.1)	16.0	(8.7, 25.4)	28.1	(17.8, 45.5)
Her2neu and trastuzumab														
Her2 -/no trastuzumab	2036	1577	4.6	(2.2, 9.1)	2.7	(1.3, 5.5)	24.6	(16.6, 36.1)	30.1	(20.3, 44.1)	14.9	(8.9, 25.7)	25.4	(14.3, 42.1)
Trastuzumab	73	30	4.3	(1.7, 9.4)	3.7	(1.0, 7.0)	26.2	(14.9, 35.6)	30.2	(24.2, 43.1)	19.8	(10.3, 30.9)	38.1	(28.0, 50.4)
Her2 + /no trastuzumab	461	330	4.8	(2.1, 9.5)	2.7	(1.3, 4.9)	23.0	(16.0, 34.5)	29.8	(19.1, 43.8)	16.5	(8.9, 27.3)	29.6	(16.1, 47.3)
Her2 or trastuzumab unknown	130	90	4.2	(2.1, 9.0)	2.7	(1.2, 5.5)	24.3	(15.5, 37.6)	29.8	(19.7, 42.7)	15.4	(8.0, 25.6)	26.2	(14.0, 40.9)
Radiotherapy														
No	643	395	3.7	(2.0, 8.7)	2.1	(1.0, 4.1)	25.5	(16.7, 37.7)	31.9	(21.3, 46.9)	15.5	(9.6, 27.5)	31.3	(21.0, 48.5)
Yes	2039	1631	4.8	(2.3, 9.4)	2.8	(1.4, 5.8)	24.2	(16.3, 35.3)	29.5	(19.6, 43.5)	15.4	(8.7, 26.1)	24.7	(13.3, 41.3)
Chemotherapy (related to blood draw)														
No CT	1340	1134	4.4	(2.2, 9.0)	2.8	(1.4, 5.8)	25.1	(17.1, 37.4)	28.5	(19.2, 42.6)	15.1	(8.8, 25.7)	20.7	(11.2, 37.7)
Yes, 1st blood draw prior CT	516	257	4.0	(1.9, 8.5)	2.7	(1.3, 5.2)	22.5	(15.7, 34.2)	32.1	(22.2, 47.0)	19.6	(11.6, 34.4)	30.2	(21.4, 46.7)
Yes, 1st blood draw during CT/ < 3 Mon. after CT	327	174	4.1	(2.1, 8.7)	2.9	(1.4, 4.8)	22.9	(15.5, 34.5)	34.8	(22.2, 52.0)	18.6	(12.6, 30.2)	33.3	(22.1, 48.6)
Yes, 1st blood draw > = 3 month after CT	483	460	5.9	(2.6, 10.7)	2.5	(1.1, 4.9)	24.6	(16.7, 35.0)	31.2	(20.3, 43.9)	11.1	(7.1, 19.2)	31.6	(20.6, 47.5)
Unknown	34	2	5.8	(2.7, 11.2)	1.2	(0.3, 2.1)	22.2	(14.1, 35.6)	53.3	(42.2, 64.4)	13.1	(7.9, 26.6)	46.3	(29.4, 63.2)
Tamoxifen or aromatase inhibitor														
No	536	371	4.0	(2.0, 9.0)	2.4	(1.2, 5.0)	25.3	(17.0, 36.4)	31.9	(21.7, 45.8)	15.8	(9.3, 29.1)	28.7	(17.7, 45.2)
Yes	2090	1643	4.8	(2.2, 9.2)	2.8	(1.3, 5.5)	24.1	(16.3, 35.8)	29.4	(19.5, 43.2)	15.4	(8.9, 26.1)	25.5	(13.9, 43.0)
Unknown	74	13	4.1	(1.6, 10.3)	2.5	(0.5, 6.9)	24.9	(16.1, 40.3)	34.7	(24.7, 57.7)	13.9	(7.9, 21.4)	20.2	(7.3, 39.4)
Previous tumor														
No	2543	1921	4.6	(2.2, 9.1)	2.8	(1.3, 5.5)	24.5	(16.6, 36.0)	30.0	(20.2, 44.0)	15.3	(8.9, 26.3)	25.6	(14.1, 42.7)
Yes	157	106	4.6	(2.1, 11.4)	2.0	(1.1, 4.3)	24.0	(15.6, 35.4)	30.1	(18.2, 45.8)	17.0	(9.9, 25.1)	36.0	(24.3, 52.0)
Menopausal hormone therapy														
No	936	573	5.2	(2.5, 11.0)	3.0	(1.4, 6.1)	23.5	(15.6, 35.8)	29.4	(18.4, 43.0)	18.0	(10.2, 30.7)	28.1	(14.3, 46.7)
Past	554	414	5.3	(2.5, 9.7)	3.0	(1.4, 6.1)	22.8	(16.0, 33.9)	29.7	(20.3, 43.5)	14.9	(9.3, 24.3)	26.2	(14.8, 44.1)
Current	1197	1030	3.8	(1.9, 7.6)	2.5	(1.2, 4.8)	25.5	(17.9, 36.6)	30.8	(20.8, 44.5)	13.7	(7.9, 24.2)	25.6	(15.1, 41.3)
Mode of detection														
Clinically or self-detected	1731	1211	4.5	(2.1, 9.4)	2.8	(1.3, 5.5)	24.2	(16.1, 35.9)	30.7	(20.6, 44.9)	16.7	(9.7, 28.4)	27.2	(15.4, 43.9)
Imaging	961	811	4.8	(2.3, 9.0)	2.6	(1.3, 5.3)	24.7	(17.1, 36.2)	29.3	(19.2, 42.4)	13.3	(7.8, 23.7)	24.7	(13.7, 42.1)
Diabetes														
No	2450	1882	4.5	(2.1, 9.0)	2.7	(1.2, 5.2)	24.9	(16.9, 36.5)	30.5	(20.6, 4.3)	15.0	(8.7, 25.7)	26.0	(14.7, 42.9)
Yes	245	143	5.7	(2.7, 11.2)	3.8	(1.9, 8.6)	18.6	(13.5, 28.4)	24.3	(14.1, 39.5)	18.0	(11.3, 33.8)	30.4	(14.4, 48.6)
CVD														
No	2210	1671	4.3	(2.1, 8.8)	2.7	(1.2, 5.2)	24.5	(16.6, 35.6)	29.9	(20.0, 43.6)	15.5	(8.9, 26.1)	25.9	(14.9, 43.1)
Yes	490	356	5.8	(2.5, 10.9)	2.9	(1.5, 6.7)	23.7	(15.7, 37.1)	31.3	(20.2, 46.3)	15.2	(8.8, 27.0)	27.5	(14.0, 44.3)
Alcohol at diagnosis														
No alcohol	631	425	5.3	(2.3, 11.2)	3.3	(1.5, 6.4)	22.4	(15.6, 35.0)	29.5	(19.4, 44.9)	17.3	(9.9, 28.9)	26.2	(15.1, 44.1)
< 19 g/day	1679	1295	4.6	(2.2, 9.0)	2.7	(1.3, 5.3)	24.7	(16.7, 36.2)	29.7	(19.6, 43.1)	15.2	(8.9, 26.0)	26.2	(14.6, 43.4)
19 + g/day	388	306	3.7	(2.0, 7.2)	2.3	(1.2, 4.6)	24.7	(16.9, 37.2)	31.3	(22.5, 45.5)	13.8	(8.0, 24.1)	26.2	(14.6, 41.2)
Smoking at diagnosis														
Never smoker	1471	1081	4.9	(2.3, 9.7)	3.0	(1.6, 5.9)	24.7	(16.8, 36.2)	30.2	(20.1, 45.2)	16.7	(9.7, 27.3)	25.1	(13.8, 40.2)
Ex-smoker	726	611	4.5	(2.2, 9.0)	2.6	(1.3, 5.1)	24.5	(16.3, 36.6)	31.6	(21.4, 43.6)	13.3	(7.8, 23.5)	26.0	(15.5, 44.0)
Current smoker	503	335	3.7	(1.6, 7.9)	1.9	(0.9, 4.3)	23.3	(15.5, 34.7)	26.6	(18.2, 40.0)	14.9	(8.8, 29.5)	29.1	(16.3, 50.9)
Leisure time PA since age 50y (quintiles of MET*h/week)														
< = 21.33	562	381	4.3	(2.1, 9.5)	3.2	(1.5, 6.8)	23.5	(15.7, 36.2)	31.2	(20.4, 45.2)	20.8	(11.7, 35.0)	25.0	(13.9, 43.4)
< = 35.04	532	408	5.2	(2.4, 10.1)	2.8	(1.3, 5.8)	24.2	(15.7, 36.7)	30.8	(19.6, 44.4)	15.4	(8.8, 25.8)	25.6	(13.6, 41.8)
< = 49.64	534	419	4.9	(2.3, 9.6)	2.6	(1.2, 5.2)	23.3	(16.9, 34.1)	29.7	(19.6, 43.3)	14.7	(9.0, 23.7)	27.3	(16.1, 44.9)
< = 71.63	538	397	4.4	(2.1, 9.0)	2.4	(1.3, 4.8)	24.6	(16.7, 36.3)	30.7	(21.5, 43.8)	14.2	(8.3, 24.7)	26.2	(14.6, 40.4)
71.64 +	504	402	4.0	(2.0, 7.6)	2.5	(1.1, 4.6)	26.0	(17.8, 36.8)	28.0	(19.2, 43.2)	13.1	(7.6, 23.0)	26.6	(15.1, 45.4)

*RNK* Rhein-Neckar Karlsruhe region; *CT* chemotherapy; *ER* estrogen receptor, *PR* progesterone receptor; *CVD* cardiovascular diseases; *PA* physical activity; *MET* metabolic equivalents.

CVD comprising angina pectoris, myocardial infarction, stroke, thrombosis, and peripheral artery disease.

Tests for proportional hazards were based on correlation of weighted Schoenfeld residuals for each variable with log-transformed failure times for all outcomes. There was no evidence of non-proportional effects in any of the biomarkers and covariates. We performed a sensitivity analysis for BCM by excluding patients who received neoadjuvant chemotherapy and had metastases, since both have the poorest prognosis. In an additional sensitivity analysis, we restricted BCM analyses to women with repeated adipokine measurements (at both baseline and follow-up), considering time-at-risk and events only after the second blood draw. Potential interaction with BMI was assessed by adding multiplicative terms for continuous BMI and each adipokine in fully adjusted models of the complete sample and by stratification at < / >  = 25 kg/m^2^. Furthermore, to assess whether effects of adipokines were mediated by BMI and vice versa, adjusted models for all outcomes were repeated without controlling for BMI or adipokines, respectively. Finally, we evaluated potential modification by age and hormone receptor status by including separate continuous adipokine variables for two age groups (< and >  = 60 years) into fully adjusted models of BCM stratified by ERPR status.

All tests were two-sided with *p* < 0.05 considered statistically significant. Analyses were calculated in SAS, Version 9.4 (SAS Institute Inc., Cary, NC, USA).

### Ethics approval and consent to participate

All study participants gave written informed consent. The ethics committee of the University of Heidelberg, the Hamburg Medical Council and the Medical Board of the State of Rhineland-Pfalz gave approval. The study was conducted in accordance with the Declaration of Helsinki.

## Results

Median follow-up time was 11 years (range 16 days–12.9 years). During this time, 649 patients died from any cause, 401 died from breast cancer and in 464 a recurrence occurred. The characteristics of the study population are shown in Table 1 stratified by all available baseline and follow-up adipokine concentrations. Supplemental Table S2 shows the changes in adipokine concentrations across the same characteristics but restricted to those subjects with repeated measurements.

Baseline adipokine concentrations varied by timing of blood draw and non-systematically in relation to chemotherapy (supplemental Table S3a and S3b). Compared to patients without chemotherapy, patients with a blood draw prior to chemotherapy had the highest median baseline resistin concentrations (supplemental Table S3b), but there was no significant difference in adipokine concentrations compared to those who had a blood draw during chemotherapy (supplemental Table S3c). Measurements of BMI, leptin, and adiponectin were highly correlated between baseline and follow-up (study region-adjusted Spearman’s *r* = 0.90, 0.58, 0.55, respectively), whereas resistin measurements were weakly correlated (*r* = 0.18). At baseline, inter-correlations between adipokines were low (all *r* <  ± 0.16), while BMI was positively correlated with leptin (*r* = 0.65) and negatively with adiponectin (*r* = −0.25).

### All-cause mortality

The basic model including mutually adjusted adipokines, age, region and time of blood draw showed that neither continuous leptin nor adiponectin (HR 1.04, 95% CI 0.99–1.10 and HR 0.94, 95% CI 0.86–1.03, respectively) or their quintiles were related to all-cause mortality (Table 2). Higher resistin concentrations were associated with increased all-cause mortality (HR 1.14, 95% CI 1.06–1.23) and the highest resistin quintile showed a significant HR 1.40 (95% CI 1.07–1.84) (Table 2). In the fully adjusted models, adipokines were not associated with all-cause mortality (Table 2).

**Table 2:** Proportional hazards analysis of all-cause mortality, breast cancer specific mortality, and recurrence with time-varying adipokines^a^.

*N*/events	All-cause mortality				Breast cancer specific mortality				Risk of recurrence			
	3,112/649	*P*	3,022/623	*P*	3,112/401	*P*	3,010/381	*P*	2,878/464	*P*	2,786/443	*P*
	HR (95% CI)		HR (95% CI)		HR (95% CI)		HR (95% CI)		HR (95% CI)		HR (95% CI)	
	Basic model^b^		Full model^c^		Basic model^b^		Full model^d^		Basic model^b^		Full model^d^	
**Leptin** continuous	1.04 (0.99, 1.10)	0.15	0.97 (0.92, 1.04)	0.42	1.03 (0.96, 1.10)	0.44	0.94 (0.86, 1.02)	0.14	1.07 (1.01, 1.14)	0.03	1.001 (0.93, 1.08)	0.98
Baseline quintiles^e^ (ng/ml)												
< 1.82	Reference		Reference		Reference		Reference		Reference		Reference	
< 3.47	0.94 (0.74, 1.18)		0.89 (0.70, 1.14)		1.13 (0.84, 1.51)		1.10 (0.80,1.50)		0.93 (0.70, 1.24)		0.89 (0.66, 1.20)	
< 5.98	0.87 (0.68, 1.11)		0.82 (0.63, 1.07)		0.97 (0.70, 1.32)		0.91 (0.65, 1.28)		1.24 (0.94, 1.63)		1.13 (0.83, 1.52)	
< 10.66	0.99 (0.77, 1.26)		0.90 (0.68, 1.19)		1.18 (0.86, 1.60)		0.99 (0.70, 1.41)		1.06 (0.79, 1.42)		0.94 (0.67, 1.32)	
10.66 +	1.30 (1.01, 1.65)		0.94 (0.70, 1.26)		1.25 (0.91, 1.73)		0.92 (0.62, 1.36)		1.41 (1.05, 1.89)		1.04 (0.72, 1.48)	
**Adiponectin** continuous	0.94 (0.86, 1.03)	0.18	0.99 (0.90, 1.08)	0.81	0.95 (0.85, 1.06)	0.33	0.98 (0.88, 1.11)	0.58	1.01 (0.91, 1.12)	0.93	1.07 (0.95, 1.19)	0.26
Baseline quintiles^e^ (mg/l)												
< 14.95	Reference		Reference		Reference		Reference		Reference		Reference	
< 21.26	0.97 (0.75, 1.25)		1.10 (0.84, 1.43)		0.84 (0.60, 1.16)		0.92 (0.66, 1.30)		1.02 (0.75, 1.38)		1.14 (0.83, 1.57)	
< 28.14	0.93 (0.72, 1.20)		1.11 (0.85, 1.45)		0.88 (0.63, 1.21)		0.98 (0.69, 1.37)		0.98 (0.72, 1.34)		1.23 (0.89, 1.70)	
< 39.31	0.83 (0.64, 1.08)		0.99 (0.76, 1.31)		0.93 (0.68, 1.28)		1.06 (0.76, 1.48)		1.16 (0.87, 1.56)		1.29 (0.94, 1.77)	
39.31 +	0.97 (0.76, 1.25)		1.16 (0.89, 1.50)		0.94 (0.69, 1.29)		1.06 (0.77, 1.47)		1.02 (0.76, 1.38)		1.23 (0.89, 1.68)	
**Resistin** continuous	1.14 (1.06, 1.23)	< 0.01	0.98 (0.90, 1.06)	0.55	1.20 (1.09, 1.32)	< 0.01	0.97 (0.88, 1.08)	0.79	1.07 (0.98, 1.16)	0.15	0.94 (0.85, 1.03)	0.17
Baseline quintiles^e^ (ng/ml)												
< 7.87	Reference		Reference		Reference		Reference		Reference		Reference	
< 12.57	1.01 (0.75, 1.36)		1.02 (0.75, 1.38)		1.04 (0.70, 1.55)		1.03 (0.68, 1.56)		0.98 (0.69, 1.38)		1.05 (0.74, 1.51)	
< 19.09	1.20 (0.90, 1.60)		1.05 (0.78, 1.41)		1.40 (0.96, 2.05)		1.17 (0.78, 1.73)		1.04 (0.74, 1.45)		0.94 (0.66, 1.35)	
< 31.39	1.09 (0.82, 1.46)		0.82 (0.61, 1.01)		1.17 (0.80, 1.73)		0.83 (0.55, 1.25)		1.02 (0.73, 1.43)		0.83 (0.58, 1.19)	
31.39 +	1.40 (1.07, 1.84)		0.93 (0.70, 1.24)		1.79 (1.25, 2.56)		1.04 (0.70, 1.53)		1.16 (0.84, 1.60)		0.90 (0.64, 1.27)	
**BMI (kg/m**^**2**^**)**												
< 22.5			1.29 (0.99, 1.67)				1.39 (1.00, 1.93)				0.97 (0.72, 1.31)	
22.5- < 25			Reference				Reference				Reference	
≥ 25- < 30			1.22 (0.96, 1.54)				1.32 (0.97, 1.80)				0.96 (0.74, 1.25)	
30 +			1.27 (0.95, 1.70)				1.30 (0.89, 1.89)				1.05 (0.75, 1.45)	

^a^Proportional hazards regression models were modified for clustered data by introducing the random laboratory batch number.

^b^Models were adjusted for continuous age, region, and time of blood draw after diagnosis.

^c^Models were adjusted for the other adipokines, BMI, region, age at diagnosis (continuously), time between diagnosis and first blood draw, timing of blood draw in relation to chemotherapy, tumor size, nodal status, metastasis, grading, combined estrogen/progesterone receptor status, previous tumors, diabetes at baseline, CVD at baseline comprising angina pectoris, myocardial infarction, stroke, thrombosis, and peripheral artery disease), MHT use, smoking, alcohol consumption, leisure time PA at age 50 (quintiles of MET x h/wk), mode of detection by imaging (yes/no).

^d^Models were adjusted as in (c) but not for diabetes at baseline, CVD at baseline, smoking, and alcohol consumption. Additionally adjusted for combined Her2 receptor status/trastuzumab use, radiotherapy, tamoxifen and/or aromatase inhibitor.

^e^Quintiles were derived from baseline measurements. FU measurements were categorized into respective baseline quintiles.

### Breast cancer specific mortality

Similar to all-cause mortality, the basic model yielded a significant association for resistin with BCM (e.g. continuous HR 1.20, 95% CI 1.09–1.32) but not for leptin and adiponectin (Table 2). In the fully adjusted model (Table 2), none of the adipokines were associated with BCM. Hazard ratios for adipokines were attenuated mainly by tumor characteristics and to a lesser extent by BMI. The sensitivity analysis after excluding primary metastasized patients and those who received neoadjuvant chemotherapy also showed lack of association (supplemental Table S4).

### Risk of recurrence

Using the basic model, the risk of recurrence was significantly increased with increasing leptin concentrations (HR 1.07, 95% CI 1.01–1.14; top quintile HR 1.41, 95% CI 1.05–1.89) but not for adiponectin or resistin (Table 2). None of the adipokines were associated with risk of recurrence in the fully adjusted model (Table 2).

### All-cause mortality, BCM and recurrence risk in patients with repeated adipokine measurement

Among women with repeated adipokine measurements (*n* = 1576), who had a median time-at-risk of 6 years after the follow-up blood draw, adipokines were not related to any of the investigated outcomes (Table 3).

**Table 3:** Associations between adipokines and all-cause mortality and breast cancer specific mortality in breast cancer patients with repeated measurements.

*N*/events	All-cause mortality^a^		Breast cancer mortality^b^		Risk of recurrence^b^	
	1,587/147		1,576/74		1,466/92	
	HR (95% CI)	*p*	HR (95% CI)	*p*	HR (95% CI)	*p*
Leptin continuous	0.98 (0.85, 1.12)	0.73	0.98 (0.80, 1.19)	0.81	1.05 (0.88, 1.25)	0.58
Baseline quintiles ^c^ (ng/ml)						
< 1.82	Reference		Reference		Reference	
< 3.47	0.99 (0.62, 1.59)		0.93 (0.46, 1.85)		0.60 (0.31, 1.14)	
< 5.98	0.82 (0.48, 1.40)		0.90 (0.42, 1.93)		1.20 (0.66, 2.18)	
< 10.66	1.10 (0.61, 1.98)		1.43 (0.63, 3.21)		0.72 (0.32, 1.59)	
10.66 +	1.43 (0.76, 2.71)		1.40 (0.53, 3.71)		0.90 (0.35, 2.28)	
Adiponectin continuous	1.17 (0.95, 1.44)	0.15	1.18 (0.89, 1.59)	0.27	1.04 (0.80, 1.36)	0.78
Baseline quintiles ^c^ (mg/l)						
< 14.95	Reference		Reference		Reference	
< 21.26	1.95 (0.92, 4.12)		1.37 (0.44, 4.33)		1.28 (0.56, 2.92)	
< 28.14	1.80 (0.86, 3.75)		2.53 (0.90, 7.12)		1.00 (0.42, 2.39)	
< 39.31	1.31 (0.62, 2.77)		1.84 (0.66, 5.12)		1.04 (0.46, 2.34)	
39.31 +	1.84 (0.91, 3.70)		1.74 (0.64, 4.73)		1.20 (0.55, 2.61)	
Resistin continuous	1.11 (0.93, 1.33)	0.26	1.23 (0.94, 1.60)	0.13	1.13 (0.88, 1.45)	0.34
Baseline quintiles ^c^ (ng/ml)						
< 7.87	Reference		Reference		Reference	
< 12.57	1.22 (0.47, 3.20)		0.40 (0.07, 2.41)		1.08 (0.32, 3.69)	
< 19.09	1.60 (0.64, 4.01)		1.67 (0.44, 6.19)		1.78 (0.56, 5.70)	
< 31.39	1.81 (0.74, 4.43)		1.26 (0.34, 4.64)		1.03 (0.31, 3.40)	
31.39 +	1.81 (0.75, 4.36)		1.87 (0.54, 6.51)		1.51 (0.48, 4.72)	

^a^Models were adjusted for the other adipokines, BMI, region, age at diagnosis, time between diagnosis and first blood draw, timing of blood draw in relation to chemotherapy, tumor size, nodal status, metastasis, grading, combined estrogen/progesterone receptor status, previous tumors, MHT use, leisure time PA at age 50 (quintiles of MET x h/wk), mode of detection by imaging (yes/no), alcohol consumption smoking, CVD, and diabetes.

^b^Models were adjusted for the other adipokines, BMI, region, age at diagnosis, time between diagnosis and first blood draw, timing of blood draw in relation to chemotherapy, tumor size, nodal status, metastasis, grading, combined estrogen/progesterone receptor status, previous tumors, MHT use, leisure time PA at age 50 (quintiles of MET x h/wk), mode of detection by imaging (yes/no), combined Her2 receptor status/trastuzumab use, radiotherapy, tamoxifen and/or aromatase inhibitor.

^c^Quintiles were derived from baseline measurements. FU measurements were categorized into respective baseline quintiles.

### All-cause mortality, BCM and recurrence risk by ERPR status

None of the adipokines were associated with all-cause mortality in patients defined by ERPR status (supplemental Table S5). Among patients with ERPR positive tumors, adipokines were not related to BCM in fully adjusted models, regardless of whether metastasized patients were included or not (Table 4 and supplemental Table S6). On the other hand, a doubling of adiponectin concentration was associated with a 37% higher hazard for BCM in patients with ERPR negative disease (HR 1.37, 95% CI 1.04–1.80), and the highest quintile associated with a significantly elevated HR of 2.51 (95% CI 1.07–5.92). The highest quintile of resistin was non-significantly associated with higher BCM (HR 2.31, 95% CI 0.87–6.08). Excluding patients with metastases did not change the estimates for adiponectin, but eliminated the association of continuous resistin with BCM in ERPR negative disease (HR 1.09, 95% CI 0.85–1.40) (supplemental Table S6). The relationship between adipokines and BCM were not significantly modified by age (< 60 years vs. >  = 60 years) in ERPR negative or ERPR positive patients, with the exception of leptin-related BCM among the latter patients. In younger ERPR positive patients leptin was associated with increased BCM (HR 1.19, 95% CI 1.01–1.41), whereas it was inversely associated in older patients (HR 0.85, 95% CI 0.76–0.95). There was no evidence that any of the adipokines were associated with risk of recurrence in either ERPR positive or negative tumors (Table 4).

**Table 4:** Associations^a^ between adipokines and breast cancer specific mortality and risk of recurrence stratified by hormone receptor status.

*N*/events	Breast cancer specific mortality				Risk of recurrence			
	ERPR positive		ERPR negative		ERPR positive		ERPR negative	
	2,311/259		390/78		2227/328		369/91	
	HR (95% CI)	*p*	HR (95% CI)	*p*	HR (95% CI)	*p*	HR (95% CI)	p
Leptin continuous	0.94 (0.85, 1.04)	0.21	0.93 (0.77, 1.11)	0.43	1.01 (0.92, 1.10)	0.83	0.91 (0.75, 1.09)	0.30
Baseline quintiles^b^ (ng/ml)								
< 1.82	Reference		Reference		Reference		Reference	
< 3.47	0.96 (0.65, 1.42)		1.24 (0.61, 2.52)		0.73 (0.50, 1.06)		0.85 (0.44, 1.64)	
< 5.98	0.75 (0.49, 1.16)		1.28 (0.58, 2.81)		1.12 (0.79, 1.60)		0.73 (0.34, 1.58)	
< 10.66	0.88 (0.57, 1.35)		1.16 (0.48, 2.79)		0.97 (0.66, 1.43)		0.70 (0.29, 1.68)	
10.66 +	0.98 (0.62, 1.57)		0.72 (0.28, 1.89)		1.14 (0.75, 1.72)		0.50 (0.20, 1.21)	
Adiponectin continuous	0.89 (0.77, 1.03)	0.11	1.37 (1.04, 1.80)	0.03	1.05 (0.92, 1.20)	0.51	1.15 (0.88, 1.49)	0.32
Baseline quintiles ^b^ (mg/l)								
< 14.95	Reference		Reference		Reference		Reference	
< 21.26	0.76 (0.50, 1.15)		1.38 (0.55, 3.48)		1.09 (0.76, 1.57)		1.57 (0.68, 3.62)	
< 28.14	0.90 (0.61, 1.34)		1.96 (0.81, 4.72)		1.08 (0.74, 1.59)		1.57 (0.72, 3.44)	
< 39.31	0.90 (0.60, 1.35)		2.07 (0.89, 4.81)		1.28 (0.89, 1.84)		1.39 (0.64, 3.05)	
39.31 +	0.84 (0.57, 1.25)		2.51 (1.07, 5.92)		1.18 (0.82, 1.71)		1.21 (0.52, 2.82)	
Resistin continuous	0.93 (0.83, 1.05)	0.25	1.18 (0.93, 1.50)	0.18	0.92 (0.82, 1.03)	0.14	1.04 (0.82, 1.33)	0.75
Baseline quintiles ^b^ (ng/ml)								
< 7.87	Reference		Reference		Reference		Reference	
< 12.57	0.77 (0.46, 1.28)		1.81 (0.65, 5.01)		1.15 (0.74, 1.69)		1.05 (0.42, 2.67)	
< 19.09	1.18 (0.74, 1.89)		1.67 (0.62, 4.56)		0.86 (0.57, 1.31)		1.68 (0.74, 3.81)	
< 31.39	0.79 (0.49, 1.28)		1.24 (0.44, 3.51)		0.73 (0.48, 1.11)		1.02 (0.43, 1.42)	
31.39 +	0.89 (0.55, 1.39)		2.31 (0.87, 6.08)		0.80 (0.53, 1.20)		1.36 (0.58, 3.21)	

^a^Models were adjusted for the other adipokines, BMI, region, age at diagnosis, time between diagnosis and first blood draw, timing of blood draw in relation to chemotherapy, tumor size, nodal status, metastasis, grading, ERPR one or both positive, previous tumors, MHT use, leisure time PA at age 50 (quintiles of MET x h/wk), mode of detection by imaging (yes/no), combined Her2 receptor status/trastuzumab use, radiotherapy, tamoxifen and/or aromatase inhibitor.

^b^Quintiles were derived from baseline measurements. FU measurements were categorized into respective baseline quintiles.

*ERPR* estrogen receptor/progesterone receptor.

### Modification and mediation by BMI

BMI did not modify the relationship between adipokines and all-cause mortality and BCM overall (*p* for interaction all > 0.05). In patients with BMI < 25 kg/m^2^, continuous leptin was inversely related to all-cause mortality (HR 0.90, 95% CI 0.81–0.99) and BCM (HR 0.82, 95% CI 0.69–0.97) (supplemental Table S7a). When stratifying for ERPR status, the modifying effect of BMI < 25 kg/m^2^ on leptin-related mortality outcomes was restricted to ERPR positive tumors (supplemental Table S7b). Considering the risk of recurrence, BMI modified the association of continuous leptin (*p* = 0.03); however, after stratifying models at BMI >  = 25 kg/m^2^, leptin was not associated with risk of recurrence in normal or overweight/obese patients (supplemental Table S7a).

In fully adjusted models for each outcome but without controlling for BMI, associations with adipokines were substantially unchanged and thus, were not mediated by BMI (supplemental Table S8a and b). Similarly, associations of BMI with all outcomes varied little, when adipokines were not included into the fully adjusted models (supplemental Table S8c).

## Discussion

Based on an analysis of a large cohort of breast cancer patients, circulating adipokines (leptin, adiponectin and resistin) measured shortly after diagnosis and/or five years later were not found to be independent prognostic factors of long-term all-cause mortality, BCM or risk of recurrence after accounting for BMI, tumor characteristics, treatment and lifestyle. There was also no association between adipokines and subsequent outcome when considering women who survived until the first follow-up and provided blood samples both at recruitment and follow-up. On the other hand, in patients with ERPR negative tumors, higher adiponectin concentrations were associated with higher BCM regardless of BMI, suggesting a potential modifying effect by hormone receptor status (*p*_heterogeneity_ = 0.01). BMI and age did not modify associations with adiponectin or resistin, however leptin was inversely related to all-cause mortality and BCM in patients with normal BMI and in older patients with positive ERPR status.

### Adiponectin

This is the first prognostic study to report a relationship between higher adiponectin concentrations and increased BCM in the ERPR negative subgroup. Studies on prognosis of breast cancer subtypes reported a lower risk of recurrence including death from breast cancer in hormone receptor negative tumors^19^ or did not find tumor tissue adiponectin levels to be associated with overall and disease free survival in triple negative breast cancers^30^. Both those studies were small, the majority of patients were premenopausal and of non-European ethnicity, making comparisons nontrivial. It was shown that high concentrations of adiponectin conferred a higher risk of mortality, and were related to cachexia^35^ and poor physical functioning in the elderly^36,37^. High adiponectin may manifest as an early indicator of worsening physical condition in elderly hormone receptor negative patients, whose treatment response might be reduced and who have a higher probability of death compared to ERPR positive patients^38^. A more specific biological pathway may involve the upregulation of receptors AdipoR1 and AdipoR2 in dendritic cells of advanced breast cancer, leading to impaired T-cell immune response^39^. However, we did not observe circulating adiponectin to be a useful prognostic marker for breast cancer overall in our study, which is in line with that of other prognostic studies^19,21,23^.

### Resistin

As with adiponectin, we did not observe any association between resistin and outcomes investigated, but high resistin levels were non-significantly associated with poorer BCM in women with ERPR negative tumors (*p* = 0.08 for heterogeneity of top quintiles in ERPR positive versus ERPR negative tumors). However, this finding might be due to chance or small sample size. There is some supportive evidence from two studies using breast cancer tissue, one of which reported gene expression levels of resistin to be higher in triple negative breast tissue compared to luminal A subtype^32^ and the other found higher resistin expression (staining score > 50% of cells) to be associated with poorer overall survival independent of hormone receptor status^31^. Higher serum resistin has also been reported to be associated with worse tumor stage as we observed in our study and overall survival^27^. Variations in sample size, menopausal status, ethnic groups/geographic region, time of blood draw, specimen and resistin laboratory assays may have contributed to the heterogeneity of results between studies. A potential effect of high resistin in patients with ERPR negative tumors could be biologically plausible. One recently hypothesized pathway considered a higher expression of the resistin receptor CAP1 in ER negative relative to ER positive breast cancer tissue^40^. However, higher CAP1 expression was associated with decreased survival independent of ER status in that study. Other *in-vitro* studies have also reported proliferative properties of this protein^41,42^.

### Leptin

We found no evidence for any associations between leptin concentrations and mortality outcomes overall. Modified associations were observed in ERPR positive patients with normal BMI and aged 60 years or older, where higher leptin concentrations related to a reduced BCM. The overall evidence for leptin as a prognostic marker is again weak and inconsistent. While some studies found leptin (peripheral or tissue concentrations) to be unrelated to BCM or recurrences^19,23,43^, others have reported increased mortality or lower disease-free survival^20,22^ with higher concentrations and the opposite associations with tumor cell counts in metastatic patients^44^ and on obesity-related cancer mortality^45^. Circulating leptin concentrations may not reflect effective tissue levels in the target organ, as has been shown in women without cancer^46^. Adding to the discrepancies, a study of leptin immunostaining of breast cancer tissues reported more recurrences and poorer survival with low or negative detection^28^.

### Strengths and limitations

Beside the large sample size and long follow-up of 11 years, a strength of our study was the repeated measurement of adipokines in half of our total study sample, enabling us to account for time-varying concentrations five years post diagnosis in the survival analyses. Furthermore, simultaneous adjustment for tumor characteristics, time of blood draw, type of treatment, region (indicating sample type) and BMI minimized the potential for residual confounding. Differential effects of baseline timing of blood draw on circulating adipokines were not directly related to chemotherapy, but were adjusted for as well.

Adipokines were most often measured in fasting blood in prognostic studies, and we used non-fasting blood samples in our analysis, which may have obscured any associations present. However, studies have shown that adipokine concentrations are similarly highly stable in both non-fasting blood up to six hours after breakfast/lunch and in fasting blood^47,48^. The average concentrations of adiponectin and resistin were comparable to published studies^9^, while leptin levels were much lower^17^. Moreover, time since last food or beverage consumption prior blood draw, which was not collected, would have been non-differential with respect to outcomes. Laboratory assays are known to be sensitive to variations in the surrounding environment, and two studies reported difficulties with reproducibility of resistin measurements over time and high CVs^12,47^. In our study, the laboratory-based quality assessment fulfilled all requirements in that most CVs were below 10%. Unlike leptin and adiponectin, resistin concentrations varied unpredictably over time, as baseline and follow-up concentrations were not correlated. We re-measured baseline and follow-up resistin in 323 individuals with a new assay kit (R-Plex human resistin) and found the weak correlation over time being confirmed (*r*_s_ = 0.20), whereas the reproducibility at each time point was high (*r*_s_ = 0.84 respectively). Thus, assuming constant resistin concentrations over the entire follow-up may have introduced potential bias in patients who had baseline measurements only. Retrospectively, we repeated the main analyses censoring these subjects at the first follow-up date of 31 December 2009 leading to fewer events and shorter follow-up time. The results for adipokines were virtually unchanged for all outcomes overall and in ERPR defined subgroups. Lastly, a limitation is that tumor tissue levels of adipokines and corresponding receptor expression data, representing locally “effective” levels, were not available, which might have contributed important information for evaluating breast cancer prognosis.

## Conclusion

Post-diagnosis peripheral adipokine concentrations of leptin, adiponectin and resistin were not associated with all-cause mortality, BCM or recurrence in ERPR positive breast cancer cases, contributing to the emerging evidence that there may be little prognostic value of circulating adipokines. In patients with ERPR negative disease, high adiponectin concentrations were associated with elevated BCM. Additional and larger epidemiological studies are warranted to investigate the potential role of adiponectin and resistin as prognostic markers, primarily in ERPR negative breast cancer, where risk of mortality is high.

## Data availability

The datasets generated and analyzed during the current study are not publicly available due to data protection rules and individual privacy but are available from the corresponding author on agreement with the principal investigators on reasonable request.

## Supplementary Information

Supplementary Information.

## Abbreviations

BCM: Breast cancer specific mortality
BMI: Body mass index
CAP1: Adenylate cyclase-associated protein 1
CI: Confidence interval
CV: Coefficient of variation
CVD: Cardiovascular disease
CT: Chemotherapy
ER: Estrogen receptor
FU: Follow-up
HH: Hamburg
HR: Hazard ratio
MHT: Menopausal hormone therapy
MSD: MesoScale discovery electrochemiluminescence platform
PR: Progesterone receptor
REMARK: Reporting recommendations for tumor marker prognostic studies
RNK: Rhein-Neckar Karlsruhe region
