# Supplementary material for: Association of circulating leptin, adiponectin, and resistin concentrations with long-term breast cancer prognosis in a German patient cohort
Source: Sci Rep. 2021 Dec 7;11:23526. doi: 10.1038/s41598-021-02958-w (PMC8651788; doi:10.1038/s41598-021-02958-w)
Supplement: Supplementary file 1 — Supplementary Information. [file 41598_2021_2958_MOESM1_ESM.pdf]

Association of circulating leptin, adiponectin, and resistin concentrations with long-term breast cancer prognosis in a German patient cohort (Obi et al.)

Supplemental Table S1. REMARK profile of the analysis of the MARIE study on prognosis of breast cancer related to adipokines

a) Patients and variables

**Study and marker**		**Remarks**
Leptin	M1	Blood concentrations
Adiponectin	M2	Analyzed log2-transformed and quintiles
Resistin	M3	all markers simultaneously
Variables at baseline and therapy		v1 = age, v2 = region, v3 = week of blood draw after diagnosis<br>v4 = BMI , v5 = tumor size, v6 = nodal status, v7 = metastasis, v8 = grading,<br>v9 = estrogen/progesterone receptor status, v10 = previous tumors,<br>v11 = diabetes at baseline, v12 = CVD at baseline, v13 = MHT use, v14 = smoking,<br>v15 = alcohol consumption, v16 = leisure time PA at age 50 (quintiles of MET x h/wk),<br>v17 = mode of detection by imaging, v18 = combined Her2 receptor status/trastuzumab<br>v19 = radiotherapy, v20 = time of blood draw in relation to chemotherapy,<br>v21 = tamoxifen and aromatase inhibitor use

**Patients**	**n**	**n events**	**Remarks**
Assessed for eligibility	3,813		*disease:* Invasive or in situ breast cancer (all stages)<br>*Patient source:* population based two regions<br>*specimen:* archived baseline and follow-up blood samples
Excluded	824		Lost to FU n=11, missing biomarker/outliers n=666, covariate unknown n=90/102<br>blood draw prior diagnosis n=12, unknown metastasis/other tumors n=15
	243		Analysis of recurrences: n=235 with neoadj. CT, primarily metastasized,<br>recurrence prior blood draw or missing (see Fig.1), covariate missing n=91
Included	3,022	623	**Overall mortality (OM)**
	3,010	381	**Breast cancer specific mortality (BCM)**
	2,786	443	**Recurrence risk**

b) Statistical analysis and survival outcomes

**Analysis**	**Patients**	**Events**	**Variables considered**	**Results/Remarks**
A1a: multivariable (basic) OM	3,112	649	M1-M3, v1 to v3	Table 2
A1b: mutivariable OM	3,022	623	M1-M3, v1 to v17, v20	Table 2
A2a: multivariable (basic) BCM	3,112	401	M1-M3, v1 to v3	Table 2
A2b: mutivariable BCM	3,010	381	M1-M3, v1-v11, v13, v16-v21	Table 2, v11, v12, v14, v15 omitted to avoid overloading the model
A2c: multivariable OM and BCM (sensitivity)			M1-M3, v1 to v17, v20/ M1-M3, v1-v11, v13, v16-v21	Metastasized and neoadj. CT excluded (suppl. Table S4)
**Subgroups**				
A2d: repeated marker measurement (all outcomes)	1,587<br>1,576<br>1,466	147<br>74<br>92	M1-M3, v1-v11, v13, v16-v21	Table 3, see text
A2e: by hormone receptor status ERPR (all outcomes)			M1-M3, v1-v11, v13, v16-v21	Ca in situ and neoadj. CT excluded, see text. Table 4 and suppl. Table S5
A2e: sensitivity for ERPR without metastasized (BCM only)				suppl. Table S6
A3a: multivariable (basic) risk of recurrence	2,878	464	M1-M3, v1 to v3	Table 2
A3b: multivariable risk of recurrence	2,786	443	M1-M3, v1-v11, v13, v16-v21	Table 2, v11, v12, v14, v15 omitted to avoid overloading the model
A3c: recurrence by hormone receptor status				
ERPR positive	2,227	328	M1-M3, v1-v11, v13, v16-v21	Table 4
ERPR negative	369	91	M1-M3, v1-v11, v13, v16-v21	Table 4
A4a: Modification by Body Mass Index (all outcomes) and by hormone receptor status			M1-M3, v1 to v17, v20/ M1-M3, v1-v11, v13, v16-v21	suppl. Table S7
A4a: Mediation by Body Mass Index (all outcomes)			No adjustment for BMI<br>Models without adipokines	suppl. Table S8a<br>suppl. Table S8b

Supplemental Figure S1. Distribution of log(2)-transformed baseline and follow-up adipokines of breast cancer patients by study center

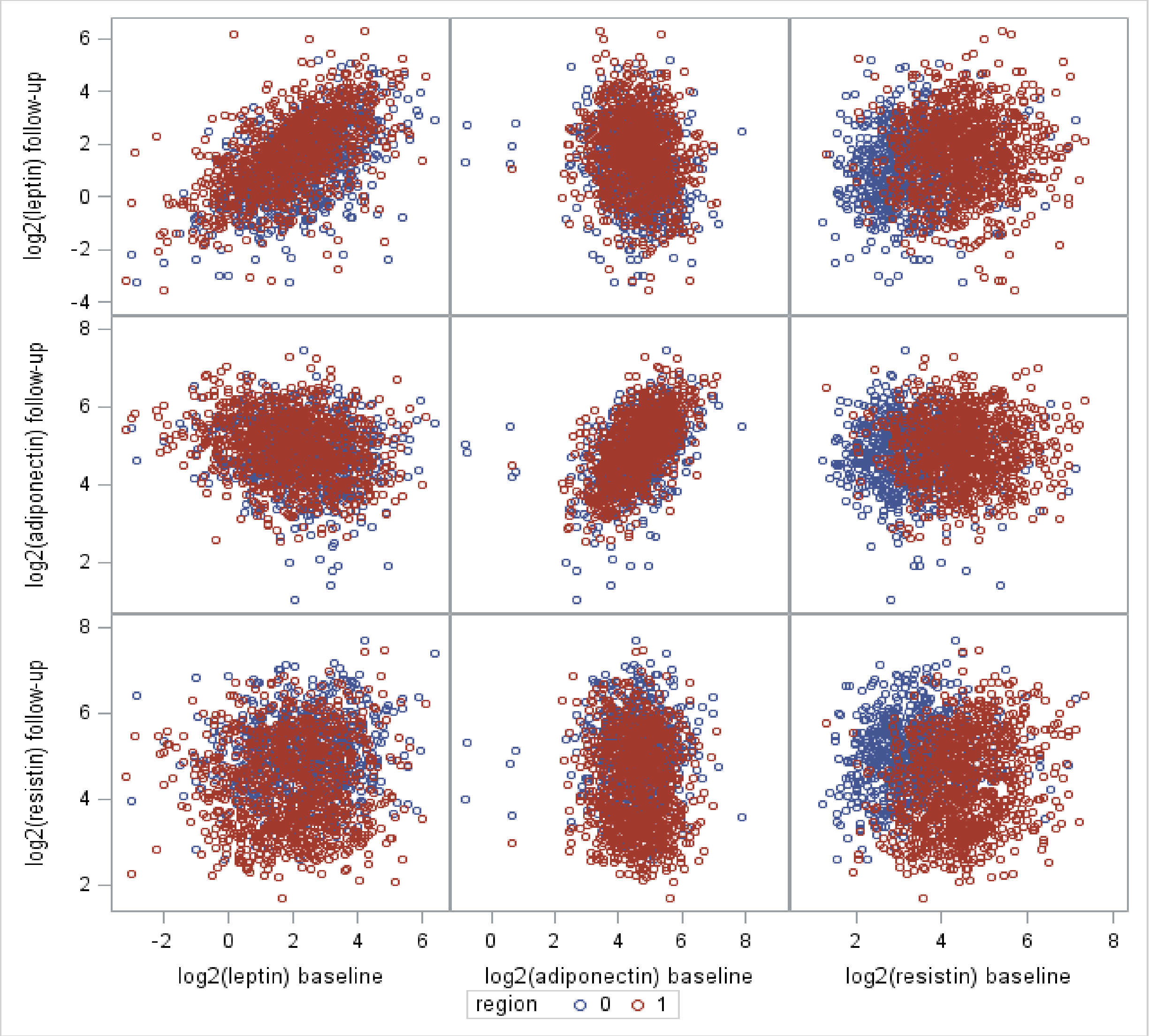

Legend: region 0= Hamburg, 1= RNK-region

Supplemental Table S2. Characteristics of patients with repeated measurement of adipokine concentrations at baseline and follow up (FU)

	Baseline & FU	Leptin (ng/ml)				Adiponectin (mg/l)				Resistin (ng/ml)			
		Baseline		FU		Baseline		FU		Baseline		FU	
	N	Median	IQR	Median	IQR	Median	IQR	Median	IQR	Median	IQR	Median	IQR
Total	1615	4.4	(2.1, 8.8)	2.8	(1.3, 5.5)	24.6	(16.6, 36.3)	30.8	(20.6, 45.2)	15.5	(9.2, 25.7)	25.9	(13.7, 42.8)
**Age**													
50 to <60 years	589	3.9	(2.0, 8.3)	2.7	(1.2, 5.0)	23.8	(16.0, 33.9)	30.8	(20.6, 43.1)	16.3	(8.8, 26.9)	27.2	(13.8, 45.5)
60+ years	1026	4.8	(2.3, 9.1)	2.8	(1.4, 5.7)	25.3	(16.8, 37.0)	30.7	(20.6, 46.1)	15.3	(9.4, 25.4)	25.2	(13.7, 41.7)
**Region**													
Hamburg	652	5	(2.3, 9.9)	2.2	(1.1, 4.3)	24.6	(17.2, 35.4)	29.2	(19.2, 41.8)	9	(6.2, 13.1)	32.7	(21.7, 49.3)
RNK region	963	4.1	(2.0, 8.1)	3.2	(1.6, 6.3)	24.7	(16.4, 36.6)	31.8	(21.3, 47.6)	22.1	(14.7, 33.3)	19.8	(10.7, 37.2)
**BMI at baseline**													
<22.5 kg/m2	357	1.8	(1.0, 3.1)	1.2	(.7, 2.2)	30.3	(21.7, 43.4)	38.4	(25.1, 53.7)	13.5	(8.1, 23.2)	25.7	(15.0, 41.7)
22.5-<25 kg/m2	433	3.3	(1.9, 6.0)	2.1	(1.2, 3.7)	25.4	(17.2, 36.8)	32.2	(21.3, 44.7)	15	(8.6, 25.3)	25	(13.3, 40.2)
25-<30 kg/m2	588	5.9	(3.6, 9.6)	3.7	(2.2, 6.4)	23	(16.0, 33.6)	28	(19.2, 42.1)	16.5	(9.7, 27.0)	25.9	(13.4, 41.5)
30+ kg/m2	236	12.2	(7.4, 18.8)	7.4	(3.9, 13.2)	20.1	(13.8, 28.1)	25.2	(17.0, 37.8)	19.1	(11.1, 30.5)	30.4	(15.0, 50.9)
**Tumorsize**													
T1 <2 cm	934	4.1	(2.0, 8.3)	2.6	(1.2, 5.4)	25.6	(16.7, 36.8)	29.4	(19.5, 44.0)	15.5	(9.0, 26.0)	23.5	(12.5, 40.9)
T2 2-5	482	4.9	(2.5, 9.9)	3.1	(1.4, 5.9)	23.2	(16.5, 34.2)	31.9	(21.4, 47.0)	15.8	(8.9, 26.1)	29.5	(17.3, 46.5)
T3 >5	40	5.9	(2.9, 13.9)	3	(1.7, 6.7)	21.3	(15.2, 25.9)	34.3	(21.2, 44.6)	18.6	(10.8, 26.6)	32.8	(19.2, 47.1)
T4 (infiltration skin or chestwall)	18	4.7	(2.6, 10.3)	3.6	(2.3, 6.5)	22.3	(18.0, 25.4)	33.2	(18.2, 41.7)	15.6	(10.4, 23.3)	32.5	(19.7, 46.4)
Neoadjuvant CT	39	5.3	(2.4, 10.5)	3.5	(2.2, 7.7)	21.2	(16.7, 35.9)	31.8	(22.5, 46.7)	17.1	(12.8, 28.9)	27.9	(18.8, 51.9)
In situ	102	3.7	(2.2, 7.2)	2.1	(1.1, 4.6)	28.9	(16.9, 42.8)	35.7	(23.1, 51.7)	12.7	(10.0, 24.1)	25.2	(15.0, 40.9)
**Nodal status**													
N0	1081	4.3	(2.2, 8.4)	2.8	(1.3, 5.7)	24.6	(16.3, 36.4)	29.4	(19.4, 43.2)	15.4	(8.9, 26.0)	23.2	(11.9, 40.2)
N1 (1-3)	285	4.6	(2.0, 9.0)	2.6	(1.2, 4.6)	25.8	(17.6, 35.6)	33.1	(22.3, 47.5)	16.3	(9.1, 26.0)	32.1	(19.0, 46.6)
N2 (4-9)	77	5.4	(2.3, 12.5)	3	(1.4, 5.3)	22.2	(16.4, 30.6)	30.8	(22.2, 43.4)	16.7	(10.1, 25.4)	35	(25.4, 44.9)
N3 (10+)	31	7.2	(5.1, 14.8)	3.4	(2.0, 9.0)	25.8	(18.2, 34.9)	44.9	(29.7, 57.9)	18.7	(9.2, 34.0)	40.1	(23.7, 54.0)
**Metastasis**													
No	1462	4.5	(2.1, 8.8)	2.8	(1.3, 5.5)	24.5	(16.6, 35.8)	30.5	(20.4, 44.7)	15.7	(9.0, 26.0)	25.9	(13.4, 43.0)
yes	7	7.9	(2.5, 13.1)	2.1	(0.7, 6.3)	24.2	(14.9, 38.9)	45.4	(13.1, 55.0)	17.1	(9.9, 25.3)	37.4	(32.4, 42.1)
unknown	5	7.6	(1.3, 13.4)	2.7	(2.3, 3.1)	30.6	(25.8, 39.4)	36.7	(25.5, 37.7)	21.2	(10.3, 52.5)	31	(12.9, 33.6)
**Grading**													
G1	320	4.2	(2.0, 8.4)	2.7	(1.4, 5.4)	26.7	(18.2, 38.1)	29.8	(19.7, 43.4)	14.7	(8.0, 26.7)	23.2	(12.4, 38.3)
G2	808	4.5	(2.2, 9.0)	2.8	(1.3, 5.8)	24.2	(16.3, 35.4)	30.3	(20.2, 44.3)	16	(9.4, 25.7)	25.1	(12.7, 42.4)
G3	339	4.8	(2.2, 8.9)	2.7	(1.3, 5.1)	23.1	(15.4, 32.9)	31.9	(21.2, 46.2)	16	(9.7, 27.6)	31.2	(17.9, 46.2)
**Hormone receptor status**	1051	4.7	(2.3, 9.0)	2.8	(1.3, 5.6)	24.5	(16.4, 35.6)	29.7	(19.8, 43.8)	15.8	(9.3, 26.9)	24.7	(12.7, 42.3)
ER+/PR+													
ER+/PR- or ER-/PR+	238	4.4	(2.0, 8.4)	2.5	(1.3, 5.0)	26.3	(18.0, 37.6)	31.9	(20.9, 45.8)	15	(8.5, 25.9)	27.2	(15.0, 42.1)
ER-/PR-	185	4	(2.1, 9.2)	2.8	(1.4, 5.7)	23.1	(15.1, 33.8)	32.2	(22.7, 47.7)	16.6	(9.1, 24.5)	29.2	(17.7, 45.5)
**Her2neu & trastuzumab**													
Her2 -/no trastuzumab	1256	4.5	(2.2, 8.8)	2.8	(1.3, 5.6)	24.7	(16.7, 36.6)	31.3	(20.9, 45.5)	15.2	(9.2, 25.6)	25	(13.4, 41.7)
Trastuzumab	22	10.3	(3.0, 13.4)	3.6	(1.0, 7.0)	21.3	(14.5, 29.8)	25.9	(23.7, 41.1)	18.1	(9.8, 23.0)	40.2	(28.1, 52.5)
Her2 +/no trastuzumab	263	4.5	(2.1, 7.9)	2.7	(1.4, 4.8)	23	(15.5, 32.4)	29.8	(19.0, 44.7)	17.1	(10.3, 27.5)	29.9	(14.6, 47.3)
Her2 or trastuzumab unknown	74	3.6	(1.8, 7.7)	2.6	(1.2, 5.7)	27.1	(19.4, 38.2)	29.8	(22.4, 46.4)	14.8	(7.6, 21.4)	25.9	(13.2, 38.5)
**Radiotherapy**													
No	312	3.5	(1.9, 7.7)	2.1	(1.0, 4.2)	26.2	(16.8, 38.4)	33.4	(22.5, 48.8)	14.6	(10.0, 24.2)	31.2	(21.4, 46.8)
Yes	1302	4.7	(2.2, 9.0)	2.9	(1.4, 5.9)	24.3	(16.5, 35.5)	30	(20.1, 44.0)	16	(8.9, 26.1)	24	(12.4, 40.9)
**Chemotherapy (related to blood draw)**													
No	922	4.2	(2.2, 8.5)	2.8	(1.4, 6.0)	25.3	(17.2, 37.8)	28.8	(19.2, 43.0)	15.6	(9.3, 26.0)	18.8	(10.5, 36.8)
First blood draw prior CT	255	3.9	(1.8, 8.1)	2.7	(1.3, 5.2)	23.3	(16.0, 34.9)	31.8	(22.0, 45.6)	18.2	(10.7, 31.1)	30.4	(21.6, 46.7)
During CT/<3 Months after CT	170	4.8	(2.2, 9.4)	3	(1.5, 4.8)	21.6	(14.7, 32.4)	34.8	(21.7, 51.7)	19.5	(13.2, 28.7)	33.5	(22.2, 49.0)
≥ 3 month after CT	266	5.3	(2.3, 10.0)	2.5	(1.1, 4.6)	24.5	(16.9, 35.2)	33	(22.4, 46.3)	11.2	(7.1, 18.2)	34.2	(21.7, 48.6)
Unknown	2	5.7	(.6, 10.9)	1.2	(.3, 2.1)	32.9	(16.7, 49.1)	53.3	(42.2, 64.4)	55.4	(4.9, 105.8)	46.3	(29.4, 63.2)
**Tamoxifen or aromatase inhibitor**													
No	298	3.8	(2.0, 8.1)	2.5	(1.3, 5.2)	25.3	(16.2, 36.2)	32.4	(22.4, 47.7)	16.6	(10.1, 28.2)	29.2	(17.7, 45.5)
Yes	1306	4.7	(2.2, 8.9)	2.8	(1.3, 5.5)	24.5	(16.6, 36.3)	30.1	(20.2, 44.2)	15.4	(9.1, 25.7)	25	(13.1, 41.8)
Unknown	11	4.9	(.9, 10.3)	3.9	(0.5, 7.3)	26	(18.2, 51.9)	34.1	(23.2, 57.7)	16.1	(8.7, 21.8)	20.2	(9.5, 39.4)
**Previous tumor**													
No	1532	4.5	(2.1, 8.8)	2.8	(1.3, 5.6)	24.6	(16.7, 36.2)	30.8	(20.7, 44.9)	15.4	(9.2, 25.7)	25.3	(13.3, 41.9)
Yes	83	4.2	(2.4, 7.7)	2.1	(1.2, 4.6)	26.4	(15.6, 38.9)	30.2	(19.9, 47.1)	16.8	(10.9, 25.7)	36.2	(26.4, 51.0)
**Menopausal hormone therapy**													
No	474	5.3	(2.6, 10.1)	3.1	(1.4, 6.1)	23.4	(15.4, 34.8)	30.6	(19.4, 44.7)	18.4	(10.5, 30.3)	27.7	(13.3, 47.3)
Past	345	5	(2.4, 9.6)	3.1	(1.4, 6.1)	23.9	(16.5, 34.8)	30	(20.5, 44.3)	15.7	(9.9, 23.4)	25.5	(13.8, 42.1)
Current	789	3.7	(1.9, 7.5)	2.5	(1.2, 4.8)	25.6	(17.9, 36.8)	31.8	(21.2, 45.8)	14.1	(8.4, 24.7)	25.5	(14.1, 40.8)
**Mode of detection**													
Clinically or self-detected	963	4.3	(2.0, 8.8)	2.8	(1.3, 5.5)	24.3	(16.3, 35.3)	31.5	(21.0, 46.4)	16.6	(9.9, 26.9)	27.1	(14.4, 44.4)
Imaging	649	4.8	(2.3, 8.8)	2.6	(1.3, 5.5)	25.1	(17.2, 37.1)	29.7	(19.8, 42.9)	14.1	(8.1, 24.2)	24.1	(13.1, 40.9)
**Diabetes**													
No	1493	4.3	(2.1, 8.6)	2.7	(1.3, 5.3)	24.9	(17.2, 36.6)	31.3	(21.0, 45.6)	15.3	(9.1, 25.4)	25.7	(13.7, 42.1)
Yes	121	5.6	(2.8, 11.3)	3.9	(2.0, 8.8)	18.6	(13.8, 29.8)	25.8	(15.5, 42.3)	18.2	(11.3, 29.8)	31	(13.8, 46.6)
**CVD**													
No	1341	4.2	(2.1, 8.5)	2.7	(1.2, 5.2)	24.6	(16.7, 36.2)	30.6	(20.6, 44.7)	15.6	(9.5, 26.1)	25.6	(13.8, 42.8)
Yes	274	5.5	(2.4, 10.5)	3	(1.6, 7.3)	25	(15.9, 36.5)	32	(20.6, 46.5)	15.1	(8.7, 24.5)	27.4	(13.3, 42.9)
**Alcohol at diagnosis**													
No alcohol	351	5.3	(2.3, 11.2)	3.3	(1.5, 6.3)	23	(15.7, 36.6)	30	(20.3, 46.2)	16.9	(9.9, 27.6)	25.3	(13.9, 44.0)
<19 g/day	1034	4.4	(2.1, 8.7)	2.7	(1.3, 5.5)	25	(16.8, 36.2)	30.5	(20.3, 44.1)	15.6	(9.3, 25.8)	25.8	(13.5, 42.8)
19+ g/day	229	3.6	(2.0, 6.9)	2.3	(1.2, 4.6)	24.7	(16.9, 37.5)	32.1	(22.8, 47.6)	14.1	(8.2, 23.8)	26	(14.1, 40.9)
**Smoking at diagnosis**													
Never smoker	883	4.8	(2.3, 9.2)	3.1	(1.5, 6.0)	24.6	(16.6, 36.3)	30.8	(20.8, 45.8)	16.7	(10.0, 25.8)	24.1	(12.8, 39.4)
Ex-smoker	472	4.5	(2.1, 8.4)	2.6	(1.3, 5.2)	25.1	(16.3, 36.9)	33	(21.6, 47.9)	13.5	(8.0, 24.1)	26.2	(14.1, 43.5)
Current smoker	260	3.3	(1.7, 7.4)	2	(0.9, 4.2)	23.9	(16.7, 33.2)	26.6	(18.2, 39.6)	15.4	(9.0, 29.5)	29.9	(16.4, 51.6)
**Leisure time PA since age 50y (quintiles of MET*h/week)**													
≤ 21.33	323	4.1	(2.0, 9.2)	3.2	(1.5, 6.8)	24.5	(16.1, 36.8)	31.4	(21.2, 46.3)	20.4	(11.9, 31.7)	24.6	(13.3, 42.5)
≤ 35.04	328	5.2	(2.4, 9.9)	2.9	(1.4, 5.9)	24.9	(15.5, 36.1)	31.5	(20.4, 46.5)	16	(9.1, 25.4)	24.6	(12.7, 41.6)
≤ 49.64	332	4.9	(2.3, 9.6)	2.7	(1.2, 5.4)	23.3	(16.4, 34.8)	30.8	(19.6, 43.3)	14.8	(9.8, 24.4)	26.6	(15.4, 42.9)
≤ 71.63	322	4	(2.0, 8.3)	2.5	(1.3, 4.8)	25.1	(17.7, 37.0)	30.4	(21.1, 45.6)	14	(8.4, 24.5)	26.4	(14.4, 40.9)
71.64+	294	3.9	(2.1, 7.0)	2.5	(1.1, 4.3)	25.8	(18.4, 36.7)	29.1	(21.1, 45.5)	13.2	(7.9, 23.1)	25.7	(13.7, 45.6)
**Time of blood draw after diagnosis**													
0-4 days	107	4.2	(2.3, 8.2)	2.3	(1.1, 4.3)	24.7	(18.4, 36.6)	30.5	(19.6, 44.0)	11.6	(8.0, 24.9)	27.8	(17.0, 44.1)
5-7 days	111	3.6	(1.5, 8.1)	2.9	(1.6, 4.8)	22.5	(16.8, 34.9)	28.8	(20.6, 40.8)	21.2	(10.2, 31.8)	22.5	(11.5, 37.2)
8-14 days	195	3.7	(1.6, 7.4)	3	(1.6, 6.1)	25.5	(15.7, 37.3)	31.3	(20.4, 45.8)	20.1	(12.8, 36.7)	22.3	(12.9, 35.1)
15-28 days	136	3.3	(1.8, 6.1)	2.7	(1.2, 5.8)	24.8	(15.8, 34.0)	31.1	(21.2, 48.5)	23.2	(15.6, 39.3)	24.5	(12.2, 41.0)
5-12 weeks	153	4.8	(2.2, 9.6)	2.8	(1.4, 5.6)	26.1	(16.5, 36.4)	31.3	(20.9, 45.5)	16.9	(9.9, 26.1)	24.7	(12.7, 38.9)
13 weeks - 6 months	180	5.3	(2.8, 9.8)	3.4	(1.6, 6.7)	25.3	(14.9, 36.5)	32.6	(19.4, 46.4)	17.8	(12.7, 27.9)	25.2	(13.0, 41.0)
7-12 months	306	4.8	(2.2, 9.8)	2.8	(1.4, 5.8)	23.9	(16.8, 34.4)	32	(22.4, 47.5)	16.2	(10.1, 24.2)	24.6	(11.5, 42.8)
13-24 months	245	4	(1.9, 8.0)	2.6	(1.1, 4.9)	25.1	(16.7, 37.0)	28.6	(20.2, 44.7)	12.1	(7.7, 20.8)	28	(13.6, 46.2)
25-36 months	104	5.5	(3.4, 9.5)	2.2	(1.2, 4.3)	24.4	(16.5, 35.5)	31.1	(20.2, 41.8)	8.6	(6.0, 13.5)	33.4	(23.3, 47.7)
4-5 years	78	6.3	(3.3, 11.6)	2.3	(1.2, 4.6)	25.6	(17.7, 36.7)	31.6	(17.0, 40.2)	7.8	(6.0, 11.6)	34.6	(24.0, 52.8)

Supplemental Table S3. Description of adipokines by time of first blood draw and timing of blood draw related to chemotherapy

A) Distribution of time of first blood draw after diagnosis

			**Leptin (ng/ml)**		**Adiponectin (mg/l)**		**Resistin (ng/ml)**	
	Baseline	FU	Baseline	FU	Baseline	FU	Baseline	FU
	N	N	Median (IQR)	Median (IQR)	Median (IQR)	Median (IQR)	Median (IQR)	Median (IQR)
0-4 days	204	108	4.5 (2.1, 8.4)	2.3 (1.1, 4.3)	24.7 (18.2, 35.3)	30.6 (20.3, 44.0)	11.4 (7.8, 21.8)	27.8 (18.1, 44.1)
5-7 days	182	112	3.9 (1.8, 8.7)	2.9 (1.6, 5.0)	22.3 (16.6, 35.6)	28.8 (20.7, 41.2)	18.7 (10.0, 31.1)	22.4 (11.0, 37.2)
8-14 days	335	196	3.8 (1.8, 8.3)	3.0 (1.6, 6.1)	23.2 (15.5, 36.2)	31.3 (20.4, 45.9)	22.3 (14.7, 38.2)	22.3 (12.9, 35.1)
15-28 days	228	136	3.5 (1.9, 7.3)	2.7 (1.2, 6.0)	23.9 (15.4, 34.8)	31.1 (21.2, 48.5)	23.4 (14.4, 41.1)	24.5 (12.2, 41.0)
5-12 weeks	264	157	4.6 (2.3, 9.5)	2.9 (1.4, 5.8)	25.1 (16.2, 36.7)	31.3 (20.9, 46.2)	16.7 (10.2, 27.9)	24.2 (12.7, 38.9)
13 weeks - 6 months	299	183	4.9 (2.5, 9.6)	3.4 (1.6, 6.6)	24.6 (16.1, 35.8)	32.5 (19.4, 46.6)	17.5 (12.2, 27.8)	25.3 (12.8, 41.1)
7-12 months	454	306	5.0 (2.3, 10.0)	2.8 (1.4, 5.8)	23.6 (16.6, 34.7)	32.0 (22.4, 47.5)	16.0 (9.9, 24.2)	24.6 (11.5, 42.8)
13-24 months	424	246	4.5 (2.0, 8.8)	2.6 (1.1, 5.2)	26.6 (17.1, 37.8)	28.7 (20.1, 44.7)	12.0 (7.2, 20.5)	27.8 (13.5, 46.2)
25-36 months	175	104	5.6 (3.1, 10.0)	2.2 (1.2, 4.3)	24.2 (16.2, 34.8)	31.1 (20.2, 41.8)	8.5 (5.8, 13.5)	33.4 (23.3, 47.7)
4-5 years	135	114	6.2 (2.8, 13.3)	2.5 (1.2, 5.3)	24.1 (17.9, 35.1)	31.8 (18.8, 39.6)	8.4 (6.1, 12.4)	32.6 (21.5, 47.3)
6-7 years	0	230		2.7 (1.4, 5.5)		28.4 (18.4, 41.5)		27.4 (18.2, 44.4)
>7 years	0	135		2.2 (0.8, 4.3)		25.9 (17.8, 37.9)		27.3 (19.5, 44.9)

IQR, interquartile range; FU, follow up

B) Timing of blood draw at baseline in relation to chemotherapy (CT)

	N*	**Leptin ng/ml**	**Adiponectin mg/l**	**Resistin ng/ml**
		Median (IQR)	Median (IQR)	Median (IQR)
No CT	1355	4.41 (2.17, 8.80)	25.24 (17.12, 37.35)	15.10 (8.80, 25.84)
Prior CT	529	3.95 (1.89, 8.50)	22.54 (15.69, 34.22)	19.55 (11.61, 34.35)
During CT / < 3 Mon. after CT	336	4.09 (2.08, 8.67)	22.94 (15.50, 34.47)	18.60 (12.63, 30.20)
>=3 Months after CT	487	5.86 (2.55, 10.71)	24.58 (16.72, 35.02)	11.09 (7.08, 19.22)
Unknown	36	5.75 (2.67, 11.21)	22.21 (14.13, 35.60)	13.06 (7.91, 26.56)

* subjects included those with missing covariate information

C) P-values for pairwise differences in means from baseline resistin across groups of time of blood draw related to chemotherapy (CT)

	Prior CT	During CT / < 3 Months after CT	>=3 Months after CT	Unknown
No CT	<.0001	0.2096	0.0008	0.2438
Prior CT		0.6566	<.0001	0.9691
During CT /< 3 Mon. after CT			<.0001	0.7651
>=3 Mon. after CT				0.0138

Supplemental Table S4. Sensitivity analysis of adipokine-related all-cause mortality and breast cancer mortality excluding patients with metastasis and/or neoadjuvant chemotherapy

	All-cause mortality		Breast cancer mortality	
N events	511		286	
	HR (95% CI)	P	HR (95% CI)	P
Leptin continuous	0.99 (0.92, 1.06)	0.75	0.94 (0.87, 1.02)	0.16
Leptin quintiles (ng/ml)				
< 1.82	Reference		Reference	
< 3.47	0.92 (0.70, 1.21)		1.15 (0.80, 1.64)	
< 5.98	0.90 (0.67, 1.21)		0.96 (0.65, 1.42)	
< 10.66	0.91 (0.67, 1.24)		1.07 (0.71, 1.60)	
10.66+	1.00 (0.72, 1.38)		1.00 (0.63, 1.56)	
Adiponectin continuous	0.99 (0.90, 1.10)	0.90	0.98 (0.86, 1.12)	0.82
Adiponectin quintiles (mg/l)				
< 14.95	Reference		Reference	
< 21.26	1.12 (0.84, 1.50)		0.95 (0.64, 1.41)	
< 28.14	1.14 (0.85, 1.54)		1.06 (0.72, 1.57)	
< 39.31	1.01 (0.75, 1.36)		1.12 (0.76, 1.64)	
39.31+	1.15 (0.86, 1.53)		1.04 (0.71, 1.52)	
Resistin continuous	0.98 (0.90, 1.06)	0.55	0.99 (0.88, 1.10)	0.80
Resistin quintiles (ng/ml)				
< 7.87	Reference		Reference	
< 12.57	1.06 (0.76, 1.47)		1.07 (0.66, 1.73)	
< 19.09	1.06 (0.77, 1.46)		1.32 (0.85, 2.04)	
< 31.39	0.88 (0.64, 1.22)		0.88 (0.56, 1.39)	
31.39+	0.93 (0.68, 1.26)		1.13 (0.73, 1.75)	

All models were adjusted for the other adipokines, BMI, region, age at diagnosis, time between diagnosis and first blood draw, timing of blood draw in relation to chemotherapy, tumor size, nodal status, grading, previous tumors, use of MHT, leisure time PA at age 50 (quintiles of MET x h/wk), mode of detection by imaging (yes/no)

All-cause mortality models were additionally adjusted for alcohol consumption, smoking, CVD, and diabetes at baseline

BCM models were additionally adjusted for combined Her2 receptor status/trastuzumab use, radiotherapy, tamoxifen and/or aromatase inhibitor

Supplemental Table S5. All-cause mortality by ERPR status

	All-cause mortality*			
	**ERPR positive**		**ERPR negative**	
N/events	2,311/444		390/115	
	HR (95% CI)	p	HR (95% CI)	p
**Leptin** continuous	0.98 (0.91, 1.06)	0.60	0.91 (0.78, 1.05)	0.21
Baseline quintiles [b] (ng/ml)				
< 1.82	Reference		Reference	
< 3.47	0.82 (0.60, 1.10)		0.57 (0.32, 1.03)	
< 5.98	0.82 (0.60, 1.13)		0.76 (0.41, 1.44)	
< 10.66	0.83 (0.59, 1.16)		0.62 (0.32, 1.23)	
10.66+	0.99 (0.69, 1.40)		0.45 (0.22, 0.91)	
**Adiponectin** continuous	0.96 (0.86, 1.07)	0.43	1.15 (0.93, 1.44)	0.20
Baseline quintiles (mg/l)				
< 14.95	Reference		Reference	
< 21.26	0.96 (0.70, 1.31)		1.14 (0.60, 2.18)	
< 28.14	1.13 (0.83, 1.54)		1.09 (0.56, 2.14)	
< 39.31	0.93 (0.67, 1.28)		1.20 (0.64, 2.23)	
39.31+	1.06 (0.78, 1.44)		1.43 (0.75, 2.73)	
**Resistin** continuous	0.97 (0.88, 1.06)	0.46	1.06 (0.88, 1.29)	0.54
Baseline quintiles (ng/ml)				
< 7.87	Reference		Reference	
< 12.57	0.85 (0.59, 1.20)		1.95 (0.93, 4.08)	
< 19.09	0.95 (0.67, 1.33)		1.82 (0.88, 3.79)	
< 31.39	0.77 (0.55, 1.09)		0.93 (0.41, 2.08)	
31.39+	0.84 (0.60, 1.16)		1.69 (0.79, 3.59)	

Abbreviations: CT, chemotherapy; ERPR, estrogen receptor/progesterone receptor

* Models were adjusted for the other adipokines, BMI, region, age at diagnosis, time between diagnosis and first blood draw, timing of blood draw in relation to chemotherapy, tumor size, nodal status, grading, previous tumors, leisure time PA at age 50 (quintiles of MET x h/wk), mode of detection by imaging (yes/no), use of MHT, alcohol consumption, smoking, CVD, and diabetes

Supplemental Table S6. Sensitivity analysis of breast cancer specific mortality* by ERPR status (patients with metastases and/or neoadjuvant CT excluded)

	**ERPR positive**		**ERPR negative**	
N/events	2241/216		379/69	
	HR (95% CI)*	p	HR (95% CI)*	p
**Leptin** continuous	0.97 (0.87, 1.09)	0.65	0.88 (0.72, 1.07)	0.19
Baseline quintiles (ng/ml)				
< 1.82	Reference		Reference	
< 3.47	1.05 (0.69, 1.62)		1.17 (0.58, 3.36)	
< 5.98	0.91 (0.57, 1.44)		1.04 (0.45, 2.38)	
< 10.66	1.10 (0.68, 1.78)		1.20 (0.45, 2.85)	
10.66+	1.20 (0.72, 2.01)		0.46 (0.16, 1.32)	
**Adiponectin** continuous	0.89 (0.76, 1.04)	0.13	1.39 (1.03, 1.86)	0.03
Baseline quintiles (mg/l)				
< 14.95	Reference		Reference	
< 21.26	0.77 (0.49, 1.19)		1.57 (0.57, 4.31)	
< 28.14	0.92 (0.60, 1.43)		2.21 (0.85, 5.76)	
< 39.31	0.87 (0.56, 1.35)		2.43 (0.97, 6.12)	
39.31+	0.85 (0.55, 1.31)		2.63 (1.05, 6.63)	
**Resistin** continuous	0.95 (0.83, 1.09)	0.45	1.09 (0.85, 1.40)	0.51
Baseline quintiles (ng/ml)				
< 7.87	Reference		Reference	
< 12.57	0.83 (0.47, 1.45)		1.96 (0.69, 5.57)	
< 19.09	1.15 (0.70, 1.89)		2.16 (0.81, 5.80)	
< 31.39	0.77 (0.46, 1.29)		1.17 (0.42, 3.31)	
31.39+	0.94 (0.58, 1.54)		1.93 (0.71, 5.21)	

Abbreviations: CT, chemotherapy; ERPR, estrogen receptor/progesterone receptor

* Models were adjusted for the other adipokines, BMI, region, age at diagnosis, time between diagnosis and first blood draw, timing of blood draw in relation to chemotherapy, tumor size, nodal status, grading, previous tumors, use of MHT, leisure time PA at age 50 (quintiles of MET x h/wk), mode of detection by imaging (yes/no), combined Her2 receptor status/trastuzumab use, radiotherapy, tamoxifen and/or aromatase inhibitor

Supplemental Table S7. **a)** Analysis of continuous adipokines for all outcomes stratified by body mass index >= 25 kg/m^2

		**BMI <25 kg/m2**<br>HR (95% CI)*	p	**BMI >= 25 kg/m2**<br>HR (95% CI)*
All-cause mortality	n events	246		377
	Leptin	0.98 (0.81, 0.99)	0.032	1.01 (0.93, 1.09)
	Adiponectin	0.98 (0.84, 1.13)		1.00 (0.89, 1.12)
	Resistin	1.01 (0.89, 1.14)		0.97 (0.88, 1.07)
Breast cancer mortality	n events	152		229
	Leptin	0.87 (0.76, 0.98)	0.028	0.96 (0.87, 1.07)
	Adiponectin	1.03 (0.88, 1.25)		0.98 (0.84, 1.14)
	Resistin	1.03 (0.85, 1.25)		0.94 (0.82, 1.08)
Recurrence risk	n events	190		254
	Leptin	1.04 (0.93, 1.12)		0.99 (0.90, 1.09)
	Adiponectin	1.05 (0.88, 1.25)		1.03 (0.89, 1.18)
	Resistin	1.01 (0.87, 1.17)		0.92 (0.81, 1.04)

b) Effect modification in patients with BMI < 25 kg/m^2. Fully adjusted models of all-cause mortality and BCM stratified by ERPR status.

		**ERPR positive & BMI <25 kg/m2**<br>HR (95% CI)*	p	**ERPR negative & BMI <25 kg/m2**<br>HR (95% CI)*	p
All-cause mortality	n events	116		65	
	Leptin	0.88 (0.71, 0.99)	0.04	0.87 (0.71, 1.07)	
	Adiponectin	0.92 (0.77, 1.11)		1.26 (0.87, 1.81)	
	Resistin	0.96 (0.82, 1.11)		1.24 (0.93, 1.65)	
Breast cancer mortality	n events	91		46	
	Leptin	0.82 (0.69, 0.97)	0.02	0.91 (0.70, 1.17)	
	Adiponectin	0.87 (0.68, 1.11)		1.83 (1.18, 1.18)	0.007
	Resistin	1.01 (0.83, 1.23)		1.21 (0.87, 1.69)	

Models were adjusted for the other adipokines, BMI, region, age at diagnosis, time between diagnosis and first blood draw, timing of blood draw in relation to chemotherapy, tumor size, nodal status, grading, previous tumors, use of MHT, leisure time PA at age 50 (quintiles of MET x h/wk), mode of detection by imaging (yes/no)

All-cause mortality models were additionally adjusted for alcohol consumption, smoking, CVD, and diabetes at baseline

BCM and recurrence risk models were additionally adjusted for combined Her2 receptor status/trastuzumab use, radiotherapy, tamoxifen and/or aromatase inhibitor

Supplemental Table S8 a) Associations between adipokines and prognostic outcomes without adjustment for BMI

	**All-cause mortality**		**Breast cancer mortality**		**Risk of recurrence**	
N/events	3.022/623		3,010/381		2,786/443	
	HR (95% CI)	p	HR (95% CI)	p	HR (95% CI)	p
Leptin continuous	0.98 (0.93, 1.03)	0.45	0.95 (0.89, 1.02)	0.15	1.01 (0.95, 1.08)	0.76
Baseline quintiles (ng/ml)						
< 1.82	Reference		Reference		Reference	
< 3.47	0.89 (0.70, 1.13)		1.09 (0.80, 1.49)		0.89 (0.66, 1.20)	
< 5.98	0.82 (0.64, 1.06)		0.92 (0.66, 1.27)		1.14 (0.86, 1.51)	
< 10.66	0.92 (0.71, 1.18)		1.02 (0.74, 1.41)		0.95 (0.70, 1.29)	
10.66+	0.97 (0.75, 1.26)		0.95 (0.68, 1.33)		1.06 (0.78, 1.45)	
Adiponectin continuous	0.99 (0.90, 1.08)	0.79	0.98 (0.87, 1.10)	0.68	1.03 (0.93, 1.14)	0.58
Baseline quintiles (mg/l)						
< 14.95	Reference		Reference		Reference	
< 21.26	1.09 (0.84, 1.42)		0.92 (0.65, 1.29)		1.11 (0.81, 1.53)	
< 28.14	1.11 (0.85, 1.45)		0.97 (0.69, 1.37)		1.20 (0.87, 1.65)	
< 39.31	0.99 (0.76, 1.30)		1.05 (0.75, 1.46)		1.26 (0.93, 1.72)	
39.31+	1.15 (0.89, 1.49)		1.05 (0.76, 1.44)		1.19 (0.88, 1.63)	
Resistin continuous	0.98 (0.91, 1.06)	0.6	0.98 (0.89, 1.08)	0.69	0.95 (0.87, 1.05)	0.32
Baseline quintiles (ng/ml)						
< 7.87	Reference		Reference		Reference	
< 12.57	1.02 (0.76, 1.39)		1.04 (0.69, 1.58)		1.07 (0.74, 1.53)	
< 19.09	1.05 (0.78, 1.41)		1.16 (0.78, 1.73)		0.94 (0.66, 1.34)	
< 31.39	0.83 (0.61, 1.12)		0.84 (0.56, 1.26)		0.83 (0.58, 1.19)	
31.39+	0.94 (0.71, 1.25)		1.05 (0.72, 1.55)		0.90 (0.64, 1.27)	

b) Breast cancer specific mortality by ERPR status

	**ERPR positive**		**ERPR negative**	
N/events	2,311/259		390/78	
	HR (95% CI)	p	HR (95% CI)	p
Leptin continuous	0.96 (0.88, 1.05)	0.34	0.88 (0.75, 1.04)	0.14
Baseline quintiles (ng/ml)				
< 1.82	Reference		Reference	
< 3.47	0.98 (0.67, 1.44)		1.12 (0.56, 2.25)	
< 5.98	0.79 (0.53, 1.18)		1.14 (0.54, 2.39)	
< 10.66	0.93 (0.63, 1.38)		0.92 (0.40, 2.13)	
10.66+	1.06 (0.71, 1.59)		0.55 (0.23, 1.35)	
Adiponectin continuous	0.88 (0.76, 1.01)	0.06	1.39 (1.07, 1.81)	0.01
Baseline quintiles (mg/l)				
< 14.95	Reference		Reference	
< 21.26	0.74 (0.49, 1.12)		1.50 (0.61, 3.72)	
< 28.14	0.88 (0.59, 1.30)		2.03 (0.84, 4.92)	
< 39.31	0.88 (0.59, 1.32)		2.24 (0.97, 5.18)	
39.31+	0.80 (0.54, 1.18)		2.70 (1.16, 6.27)	
Resistin continuous	0.95 (0.84, 1.07)	0.36	1.15 (0.91, 1.45)	0.25
Baseline quintiles (ng/ml)				
< 7.87	Reference		Reference	
< 12.57	0.79 (0.47, 1.32)		1.98 (0.72, 5.41)	
< 19.09	1.21 (0.76, 1.94)		1.77 (0.65, 4.83)	
< 31.39	0.81 (0.50, 1.30)		1.39 (0.49, 3.92)	
31.39+	0.92 (0.58, 1.45)		2.27 (0.86, 5.98)	

c) Comparison of prognostic associations of body mass index with and without adjustment for adipokines

	**All-cause mortality**		**BCM**		**Recurrence risk**	
BMI (kg/m^2)	Table 2 (manuscript)	without adpokines	Table 2 (manuscript)	without adpokines	Table 2 (manuscript)	without adpokines
	HR (95% CI)	HR (95% CI)	HR (95% CI)	HR (95% CI)	HR (95% CI)	HR (95% CI)
<22.5	1.29 (0.99, 1.67)	1.34 (1.04, 1.73)	1.39 (1.00, 1.93)	1.41 (1.01, 1.95)	0.97 (0.72, 1.31)	0.98 (0.73, 1.32)
22.5-<25	Reference	Reference	Reference	Reference	Reference	Reference
≥ 25-<30	1.22 (0.96, 1.54)	1.18 (0.94, 1.48)	1.32 (0.97, 1.80)	1.26 (0.94, 1.69)	0.96 (0.74, 1.25)	0.96 (0.75, 1.24)
30 +	1.27 (0.95, 1.70)	1.23 (0.95, 1.60)	1.30 (0.89, 1.89)	1.19 (0.86, 1.67)	1.05 (0.75, 1.45)	1.02 (0.76, 1.37)

*All models were fully adjusted as listed in Table 2 and 4 (manuscript) excluding BMI (a and b) or excluding adipokines (c).
